# Psmb8 inhibits mitochondrial fission and alleviates myocardial ischaemia/reperfusion injury by targeting Drp1 degradation

**DOI:** 10.1038/s41419-024-07189-1

**Published:** 2024-11-08

**Authors:** Hui-Xiang Su, Luo-Luo Xu, Pang-Bo Li, Hai-Lian Bi, Wen-Xi Jiang, Hui-Hua Li

**Affiliations:** 1 Department of Emergency Medicine, Beijing Key Laboratory of Cardiopulmonary Cerebral Resuscitation, Beijing Chaoyang Hospital, Capital Medical University, Beijing, China; 2 Institute of Cardiovascular Diseases, First Affiliated Hospital of Dalian Medical University, No.193, Lianhe Road, Xigang District, Dalian, China

**Keywords:** Myocardial infarction, Mechanisms of disease

## Abstract

The mitochondrial dynamic imbalance is an important cause of myocardial ischaemia/reperfusion (I/R) injury and dysfunction. Psmb8, as one of the immunoproteasome catalytic subunits, is a key regulator of protein homoeostasis, inflammation and some cardiac diseases. Here, we found that the expression level and activity of Psmb8 were significantly reduced in the heart of I/R mice and in subjects with myocardial infarction (MI). Cardiomyocyte-specific Psmb8 overexpression in mice markedly ameliorated I/R-mediated cardiac injury and dysfunction, which was accompanied by reduced mitochondrial division via the downregulation of dynamin-related protein-1 (Drp1). However, Psmb8 knockout (KO) mice exhibited the opposite changes. The effects of Psmb8 on mitochondrial fission and apoptosis was confirmed in primary cardiomyocytes with overexpression or knockdown of Psmb8 in vitro. Mechanistically, Psmb8 was directly associated with Drp1 and enhanced its degradation, which subsequently suppressed I/R-mediated mitochondrial fission and cardiac injury. Conversely, knockdown of Drp1 in Psmb8-KO mice restored I/R-induced cardiac dysfunction and mitochondrial dynamic imbalance. Our study identified a new cardioprotective role of Psmb8 in cardiac I/R damage through targeting Drp1, and highlight that increasing Psmb8 activity may constitute a promising therapy for ischaemic heart disease.

## Introduction

Ischaemic heart disease has become the main risk factor for many serious heart diseases and death worldwide. Early maintenance of coronary patency by thrombolytic treatment or/and angioplasty is the most effective approach for limiting ischaemic injury; however, reperfusion can cause further cardiac cell death and dysfunction, which is termed ischaemia/reperfusion (I/R) injury [1, 2]. Accumulating evidence indicates that mitochondrial dysfunction is a central mechanism in the pathophysiology of myocardial I/R damage [3, 4]. Cardiac function is highly dependent on the energy generated by mitochondria, and the mitochondrial morphology and function was controlled through continuous fusion and fission cycles (also known as mitochondrial dynamics) [3]. Mitochondrial dynamics are tightly modulated by members of the dynamin GTPase family, which includes dynamin-related protein 1 (Drp1), a key driver of mitochondrial fission, and mitofusin-1/2 (Mfn1/2) and optic atrophy 1 (OPA1), which join mitochondrial membranes to promote mitochondrial fusion [3, 4]. Recent evidence has revealed that mitochondrial homoeostasis is critical for cardiac metabolism and performance, and defects in mitochondrial dynamics participate in the pathogenesis of myocardial I/R injury and myocardial infarction (MI) [3, 4]. Thus, proper balance of mitochondrial dynamics is crucial for preventing cardiovascular diseases, especially myocardial I/R injury.

The ubiquitin (Ub)-26S proteasome system is a major pathway that controls the degradation of misfolded and damaged proteins in eukaryotic cells [5]. The 26S proteasome complex is composed of two major subunits: a 20S proteolytic core (CP) and a 19S regulatory particle. The CP is composed of 28 subunits, which include two outer α rings and two inner β rings. Among the 14 β subunits, the three constitutive subunits β1 (PSMB6), β2 (PSMB7), and β5 (PSMB5) possess proteolytic caspase-like, trypsin-like, and chymotrypsin-like activity, respectively. In response to proinflammatory signals, such as cytokines, the three β immunosubunits β1i (Psmb9, LMP2), β2i (Psmb10, LMP10), and β5i (Psmb8, LMP7) are highly expressed and replace the three constitutive β subunits to generate the 20S immunoproteasome [5, 6]. Early experiments reported that these immunosubunits were mainly involved in MHC II antigen presentation and inflammatory diseases [5]. Recently, our data and those of other studies have revealed that immunosubunits, particularly Psmb10 (β2i) and Psmb8 (β5i), are highly expressed in the cardiovascular tissues of mice and patients with hypertension and participate in the pathogenesis of hypertensive cardiovascular disorders, such as cardiac remodelling, heart failure, atrial fibrillation (AF), abdominal aortic aneurysm (AAA) and retinopathy in mice [7–12]. Interestingly, several studies have shown that the constitutive subunit Psmb5 (β5) and immunosubunits, including Psmb10 (β2i) and Psmb8 (β5i), and the 11S activator PA28α, also regulate the pathogenesis of cardiac and cerebral I/R injury [13–16]. More recently, we found that Psmb10 (β2i) mediated protection against cardiac I/R damage in animals [17, 18]. However, the causal roles of other catalytic subunits, particularly Psmb8, in myocardial I/R injury through the regulation of mitochondrial dynamics have largely been unexplored.

Here, we provide new evidence that the Psmb8, was the most downregulated gene of the proteasome catalytic subunits in I/R hearts of mice and hypoxia/reperfusion (H/R)-treated cardiomyocytes as well as patients with MI. Our data demonstrated that increased Psmb8 level significantly ameliorated I/R-induced cardiac dysfunction, infarct area, myocyte apoptosis, and oxidative stress, possibly by maintaining mitochondrial dynamic balance via targeting Drp1. Thus, we discover a new regulatory pathway in myocardial I/R injury involving Psmb8-Drp1-mitochondrial fission and show that increasing Psmb8 expression may constitute a novel therapy for myocardial I/R injury.

## Results

### Psmb8 expression is reduced in mice with I/R and patients with MI

**Fig. 1:**
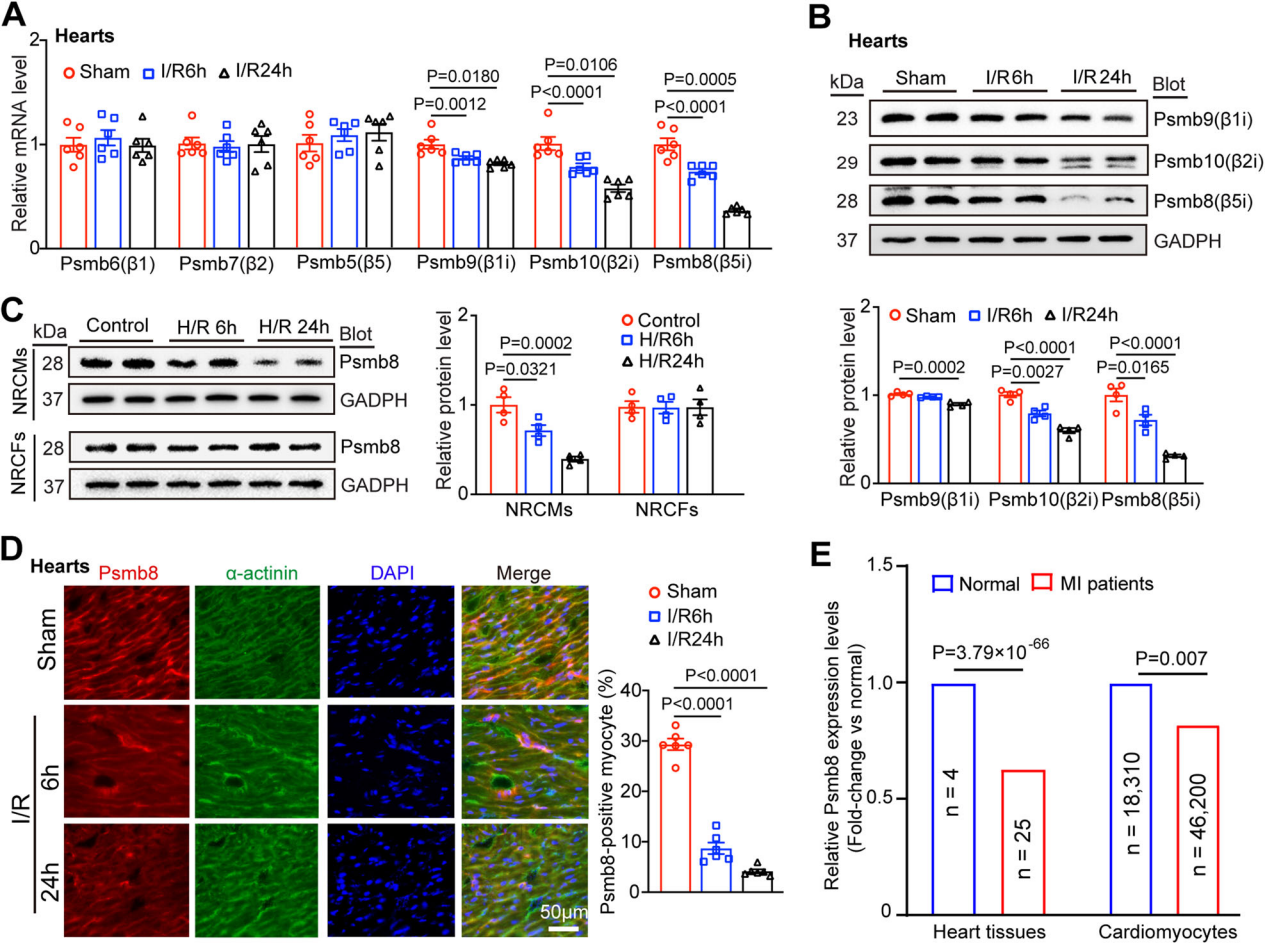
Upregulation of Psmb8 expression and activity in mice with I/R and patients. **A** qPCR analysis of the mRNA levels of proteasomal catalytic subunits (Psmb6, Psmb7, Psmb5, Psmb9, Psmb10, and Psmb8) in the board zone of the heart (*n* = 6). **B** Immunoblot analysis of Psmb9, Psmb10, and Psmb8 protein levels in the border zone of the heart (left) and quantification of the protein levels (right, *n* = 4). **C** Neonatal rat cardiomyocytes (NRCMs) and neonatal rat cardiac fibroblasts (NRCFs) were exposed to hypoxia/reoxygenation (H/R) for 6 or 24 h. Immunoblots analysis of Psmb8 protein levels in H/R-induced NRCMs (left) and the quantification (right, *n* = 4). **D** Immunofluorescence colocalization staining of heart slices with anti-Psmb8 and α-actinin antibodies (left) and quantification of the Psmb8^+^ area (right, *n* = 6). **E** The fold-change of Psmb8 expression (relative to normal) in heart tissues and cardiomyocytes. The values are expressed as the mean ± SEM, and n indicates the sample number per group. *P < 0.05 and **P < 0.01 versus the sham or control group.

To determine the involvement of the proteasome in myocardial I/R injury, we first examined the expression of six catalytic subunits (Psmb6, Psmb7, Psmb5, Psmb9, Psmb10, and Psmb8) of the proteasome in heart tissues at 6 and 24 h after I/R stress. qPCR analysis revealed that I/R did not significantly influence the mRNA levels of three standard subunits (Psmb6, Psmb7, and Psmb5) but markedly reduced the expression levels of the three immunosubunits (Psmb9, Psmb10, and Psmb8) in the heart at different time points compared with those in sham hearts (Fig. 1A). Immunoblotting further verified the decreases in Psmb9, Psmb10 and Psmb8 at the protein level in the I/R-induced hearts (Fig. 1B). Notably, the reduction in Psmb8 expression was the most significant in I/R-induced hearts (Fig. 1A, B). To further identify which cell types in the heart highly express the Psmb8 subunit, we isolated neonatal rat cardiomyocytes (NRCMs) and cardiac fibroblasts (NRCFs) and subjected them to simulated hypoxia/reoxygenation (H/R) for 6 and 24 h, respectively. Psmb8 protein levels were decreased in a time-dependent manner in NRCMs but not in NRCFs (Fig. 1C). In addition, immunostaining revealed the colocalization of Psmb8 in cardiomyocytes, and its expression was reduced in the heart after I/R injury (Fig. 1D), suggesting that I/R specifically downregulated Psmb8 expression in cardiomyocytes.

To identify whether decreased Psmb8 expression also appears in human with myocardial infarction (MI), we further analyzed published single-nucleus RNA sequencing (snRAN-seq) data [19]. Human LV samples included 4 normal hearts (with a total of 41,663 cells, of which 18,310 are cardiomyocytes) and 25 MI hearts (with a total of 150,132 cells, of which 46,200 are cardiomyocytes). The snRAN-seq analysis revealed that Psmb8 expression levels were significantly donwregulated in both tissues (Fold-change = 0.63) and cadiomyocytes (Fold-change = 0.82) compared with normal controls (Fig. 1E). Collectively, these results indicate that a marked reduction in Psmb8 expression may have a central role in the pathogenesis of myocardial I/R injury.

### Cardiac-specific Psmb8 overexpression improves I/R-induced myocardial infarction and dysfunction

**Fig. 2:**
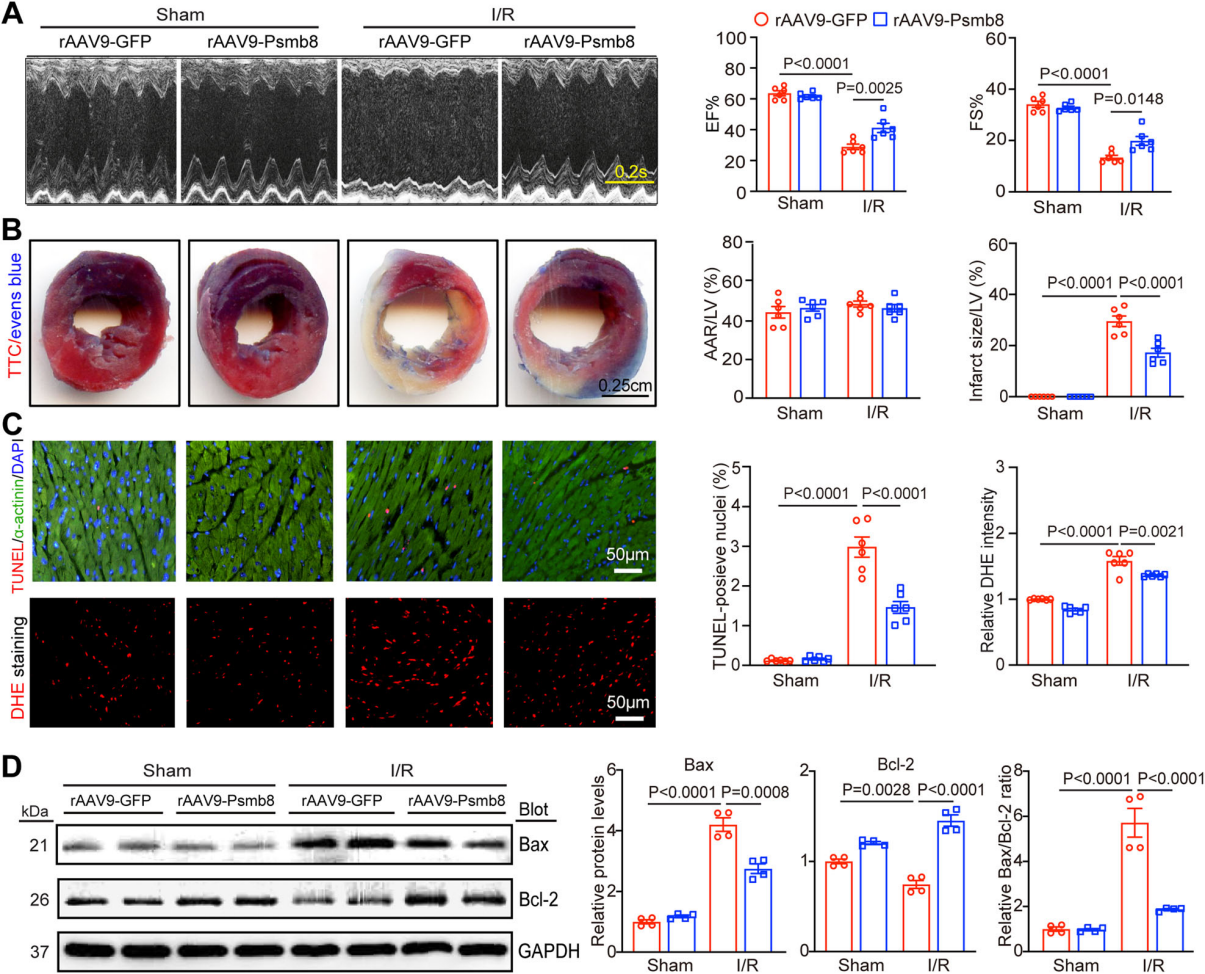
Overexpression of Psmb8 in cardiomyocytes improves I/R-mediated cardiac injury and dysfunction. **A** Male WT mice were infected with rAAV9-Psmb8 or rAAV9-GFP control for 3 weeks and then subjected to I/R or sham surgery for 24 h. Echocardiographic examination of the left ventricle (LV) (left) and percentages of the ejection fraction (EF) and fraction shortening (FS) (right, *n* = 6) are shown. **B** Images of heart sections stained with TTC and Evans blue dye (left). Percentages of the area at risk (AAR) to the LV area or the infarct area to the LV area (right, *n* = 6). Bar: 2.5 mm. **C** Images of heart sections stained with TUNEL (red), anti-α-actinin (green) and DAPI (blue) (top, left) and the percentages of TUNEL-positive nuclei (middle, *n* = 6). Heart sections were stained with DHE dye (bottom, left), and ROS concentrations were quantified (right, *n* = 6). Bar: 50 μm. **D** Immunoblot analysis of Bcl-2 and Bax protein levels (left) and quantification (right, *n* = 4). The values are expressed as the mean ± SEM, and n indicates the sample number per group.

To investigate the role of Psmb8 in the progression of myocardial I/R injury in vivo, we first injected rAAV9-Psmb8 into WT mice to specifically overexpress Psmb8 in cardiac myocytes. rAAV9-GFP was injected as a control. Three weeks after rAAV9 injection, the mice were subjected to myocardial I/R injury for 24 h. Immunoblotting revealed the Psmb8 protein levels in the hearts of the rAAV9-Psmb8-injected mice were approximately 1.7–2.5-fold higher than those in the hearts of rAAV9-GFP-injected mice following sham or I/R injury (Fig. S1A, B). Furthermore, echocardiography showed that I/R significantly reduced cardiac contractile function, as indicated by the left ventricle (LV) EF% and FS%, in rAAV9-GFP-injected mice compared with sham mice, but this decrease was restored in rAAV9-Psmb8-injected mice after I/R (Fig. 2A, Table S1). Then, we measured the cardiac infarct size 24 h after reperfusion. TTC/Evans blue staining revealed that the infarct area, as indicated by the infarct area-to-LV ratio, was significantly smaller in rAAV9-Psmb8-injected mice than in rAAV9-GFP-injected controls following I/R injury, although the area at risk (AAR)-to-total LV area ratio was the same in the I/R and sham groups (Fig. 2B). Given that apoptosis and oxidative stress are hallmarks of myocardial I/R injury, we then performed TUNEL and dihydroethidium (DHE) staining and immunoblot analysis, and the results showed that the percentage of TUNEL--positive apoptotic myocytes, superoxide levels, and the Bax-to-Bcl-2 ratio were reduced in the hearts of rAAV9-Psmb8-injected mice compared to rAAV9-GFP-injected mice after I/R injury (Fig. 2C, D). Together, overexpression of Psmb8 in cardiomyocytes prevents I/R-induced cardiac injury and dysfunction.

### Overexpression of Psmb8 in cardiomyocytes blocks I/R-induced myocardial mitochondrial fission and dysfunction

**Fig. 3:**
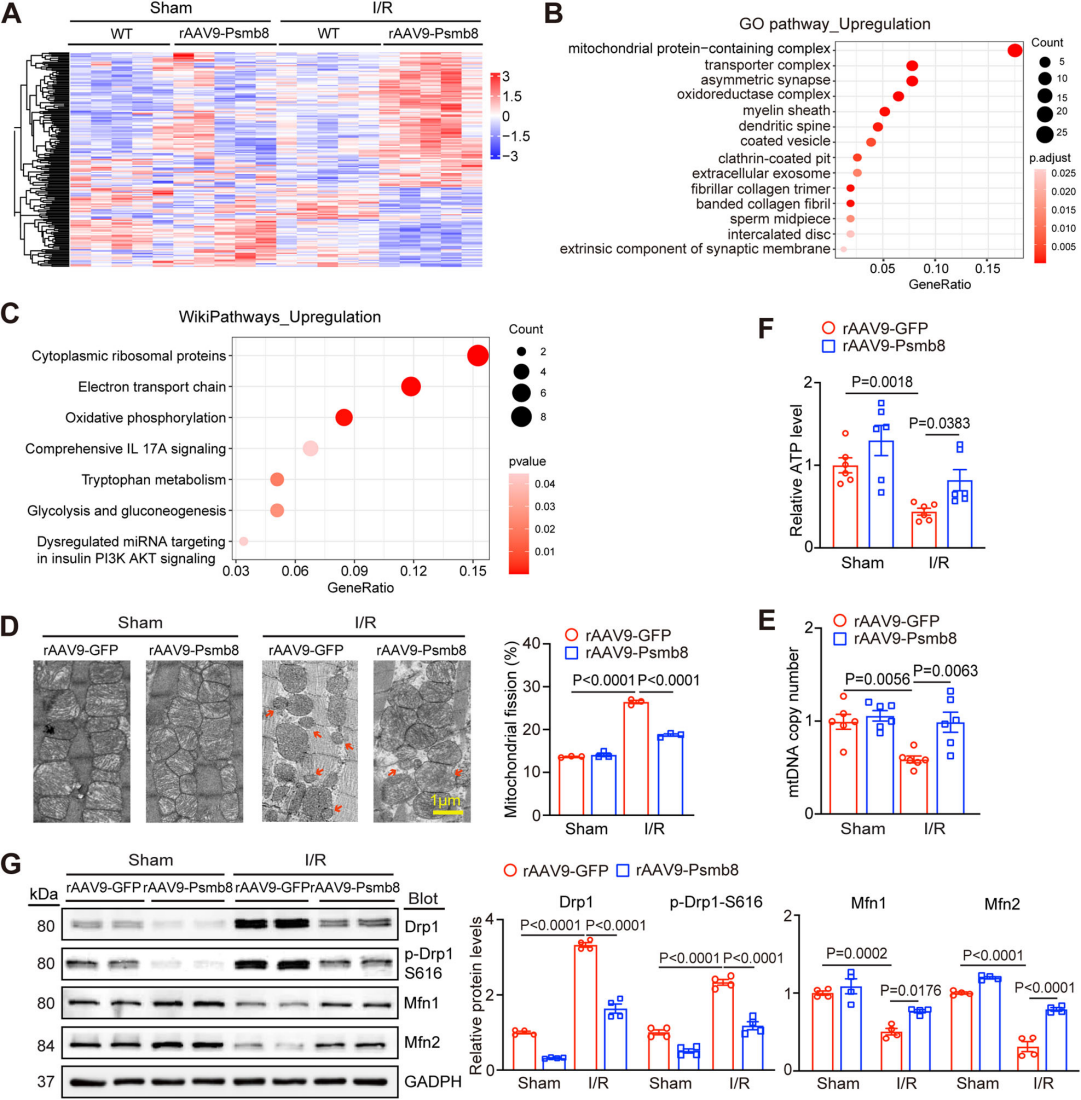
Overexpression of Psmb8 in cardiomyocytes blocks I/R-induced myocardial mitochondrial dynamic imbalance and dysfunction. **A** Heatmap of 268 differentially expressed proteins (DEPs) identified by LC–MS/MS between WT and rAAV9-Psmb8-injected mice after sham or I/R injury (*n* = 5 per group). Red: upregulated; blue: downregulated. **B** Gene Ontology (GO) functional enrichment analysis of the upregulated proteins in rAAV9-Psmb8-injected hearts. **C** Wikipathway analysis of the upwnregulated proteins in rAAV9-Psmb8 hearts. **D** Evaluation of mitochondrial morphology in cardiomyocytes using transmission electron microscopy (left). Bar: 1 µm. Red arrows indicate fragmented mitochondria. Quantification of fragmented mitochondria (right, *n* = 3). **E** qPCR analysis of NADH dehydrogenase subunit 1 to determine the mtDNA copy number per cell in the heart. The β-globin gene was used as the control. **F** Analysis of ATP levels in the hearts were measured (*n* = 6). **G** The protein levels of total Drp1, p-Drp1 (S616), Mfn1, and Mfn2 in the heart were examined by immunoblot analysis (left), and the levels of these proteins were quantified (right, *n* = 4). The values are expressed as the mean ± SEM, and n indicates the sample number per group.

To understand how Psmb8 overexpression improves cardiac I/R injury and dysfunction, we used mass spectrometry to analyse rAAV9-GFP- and rAAV9-Psmb8-injected mice following sham or I/R stress, and found that 167 proteins were upregulated and 101 proteins were downregulated in Psmb8-rAAV9-Psmb8-treated mouse hearts compared with those in WT hearts following I/R (Fig. 3A). We then assessed the differentially expressed proteins (DEPs) by GO enrichment and Wikipathway analysis, which revealed that mitochondria-related pathways, such as mitochondria-containing protein complexes, transporter complexes, oxidoreductase complexes, the electron transport chain, and oxidative phosphorylation, were significantly upregulated in the hearts of rAAV9-Psmb8-injected mice compared with rAAV9-GFP-injected mice (Fig. 3B, C, Table S2). To further evaluate the impact of Psmb8 overexpression on mitochondrial structure and function, we performed transmission electron microscopy (TEM). Our results indicated that I/R injury significantly increased the percentage of fragmented mitochondria in WT hearts compared with sham hearts, and these effects were markedly attenuated in the heart tissues of rAAV9-Psmb8-injected mice (Fig. 3D). In addition, mtDNA copy numbers and ATP levels were significantly higher in rAAV9-Psmb8-injected heart tissue than in rAAV9-GFP-injected heart tissue (Fig. 3E, F). However, the indicators of mitochondrial morphology and energy production were similar between the WT and Psmb8-TG groups following sham treatment (Fig. 3A–F). Thus, we concluded that overexpression of Psmb8 in cardiomyocytes improved mitochondrial fission and dysfunction induced by I/R.

Ample evidence has demonstrated that maintaining the balance of mitochondrial dynamics is a key mechanism for preventing cardiomyocyte apoptosis and oxidative stress during I/R injury and is tightly modulated by Drp1, Mfn1, and Mfn2. We therefore tested the effect of Psmb8 overexpression on the levels of these proteins. Immunoblot analysis indicated that I/R injury dramatically increased Drp1 and phosphorylated (p)-Drp1 (S6161) protein levels and decreased Mfn1 and Mfn2 protein levels, and these changes were reversed in rAAV9-Psmb8-injected mice following I/R (Fig. 3G). Interestingly, the I/R-induced increase of Drp1 receptor proteins (MFF, MID49, and MID51) in WT hearts was not affected in rAAV9-Psmb8-injected mice (Fig. S2A). Therefore, overexpression of Psmb8 cardiomyocytes attenuates excessive mitochondrial fission by maintaining the Drp1-Mfn1/2 balance.

### Knockout of Psmb8 exacerbates I/R-induced myocardial dysfunction, infarction, apoptosis, oxidative stress and mitochondrial fission

**Fig. 4:**
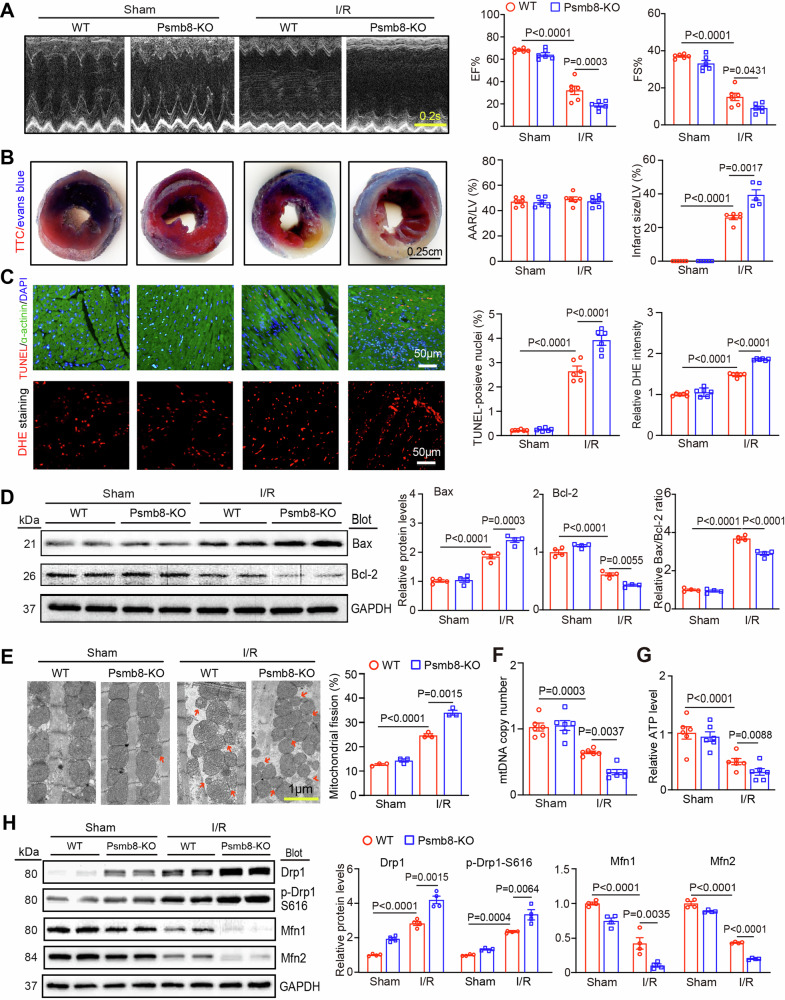
Knockout of Psmb8 accelerates I/R-induced cardiac dysfunction and injury. **A** Male WT and Psmb8-KO mice were exposed to I/R or sham conditions for 24 h. Echocardiographic examination of the left ventricle (LV) (left) and percentages of the ejection fraction (EF) and fraction shortening (FS) (right, *n* = 6) are shown. **B** Images of heart sections stained with TTC and Evans blue dye (left). Percentages of the area at risk (AAR) to the LV area or the infarct area to the LV area (right, *n* = 6). Bar: 2.5 mm. **C** Images of heart sections stained with TUNEL (red), anti-α-actinin (green) and DAPI (blue) (top, left) and the percentages of TUNEL-positive nuclei (middle, *n* = 6). Heart sections were stained with DHE dye (bottom, left), and ROS concentrations were quantified (right, *n* = 6). Bar: 50 μm. **D** Immunoblot analysis of Bcl-2, Bax and cleaved caspase-3 protein levels (left) and quantification (right, *n* = 4). **E** Mitochondrial morphology in cardiomyocytes was analysed by transmission electron microscopy (left). Bar: 1 µm. Red arrows indicate fragmented mitochondria. Quantification of fragmented mitochondria (right, *n* = 3). **F** qPCR analysis of NADH dehydrogenase subunit 1 to determine changes in mitochondrial (mt) DNA copy numbers in the heart. The β-globin gene was used as the control. **G** ATP levels in the heart were measured (*n* = 6). **H** The protein levels of total Drp1, p-Drp1 (S616), Mfn1, and Mfn2 in the heart were examined by immunoblot analysis (left), and the levels of these proteins were quantified (right, *n* = 4). The values are expressed as the mean ± SEM, and n indicates the sample number per group.

To verify the causative relationship between Psmb8 and myocardial I/R damage, we studied cardiac function and histology in Psmb8 knockout (Psmb8-KO) mice as described previously [7]. After 24 h of sham or I/R surgery, the mRNA and protein expression of Psmb8 was markedly lower in Psmb8-KO mice than in wild-type (WT) mice (Fig. S3A, B). Echocardiography indicated that cardiac performance (EF% and FS%) was lower in Psmb8-KO mice than in WT controls 24 h after reperfusion (Fig. 4A, Table S3). Moreover, the infarct area-to-LV ratio, TUNEL-positive cardiomyocytes and superoxide levels were remarkably higher in Psmb8-KO mice than in WT mice after I/R injury (Fig. 4B, C). Accordingly, apoptosis-related marker, such as the Bax-to-Bcl-2 protein ratio, was increased in Psmb8-KO mice compared with the WT controls following I/R injury (Fig. 4D), indicating that Psmb8-KO exerts proapoptotic and prooxidant effects on cardiomyocytes to accelerate myocardial I/R injury. Consistently, the I/R-induced increase in fragmented mitochondrial numbers (%) and decreases in mitochondrial (mt) DNA copy number per cell and ATP contents in WT hearts were obviously enhanced in Psmb8-KO hearts (Fig. 4E–G). Accordingly, the I/R-mediated upregulation of total and p-Drp1 protein levels and downregulation of Mfn1/2 protein levels in WT hearts were reversed in Psmb8-KO mice (Fig. 4H). However, Psmb8-KO did not altered the protein levels of MFF, MID49, and MID51 in the hearts of mice after sham or I/R surgery (Fig. S2B). Also, we examined the other targets of Psmb8 in I/R hearts [17, 18] and found that I/R-induced upregulation of PP2A and Keap1 proteins in Psmb8-KO mice was further significantly enhanced in WT hearts (Fig. S3C). The parameters for cardiac function, histopathological changes and mitochondrial dynamics were similar between WT and Psmb8-KO mice under basal conditions, although the protein levels of Drp1 and p-Drp1 were increased and levels of Mfn1/2 proteins in Psmb8-KO mice compared with WT mice after sham surgery (Fig. 4A–H). Taken together, these findings demonstrate that Psmb8 deficiency exacerbates I/R-mediated cardiac injury and dysfunction associated with increased mitochondrial fission by disrupting the Drp1-Mfn1/2 balance.

### Overexpression of Psmb8 inhibits H/R-induced apoptosis, mitochondrial fission and Drp1 expression in vitro

**Fig. 5:**
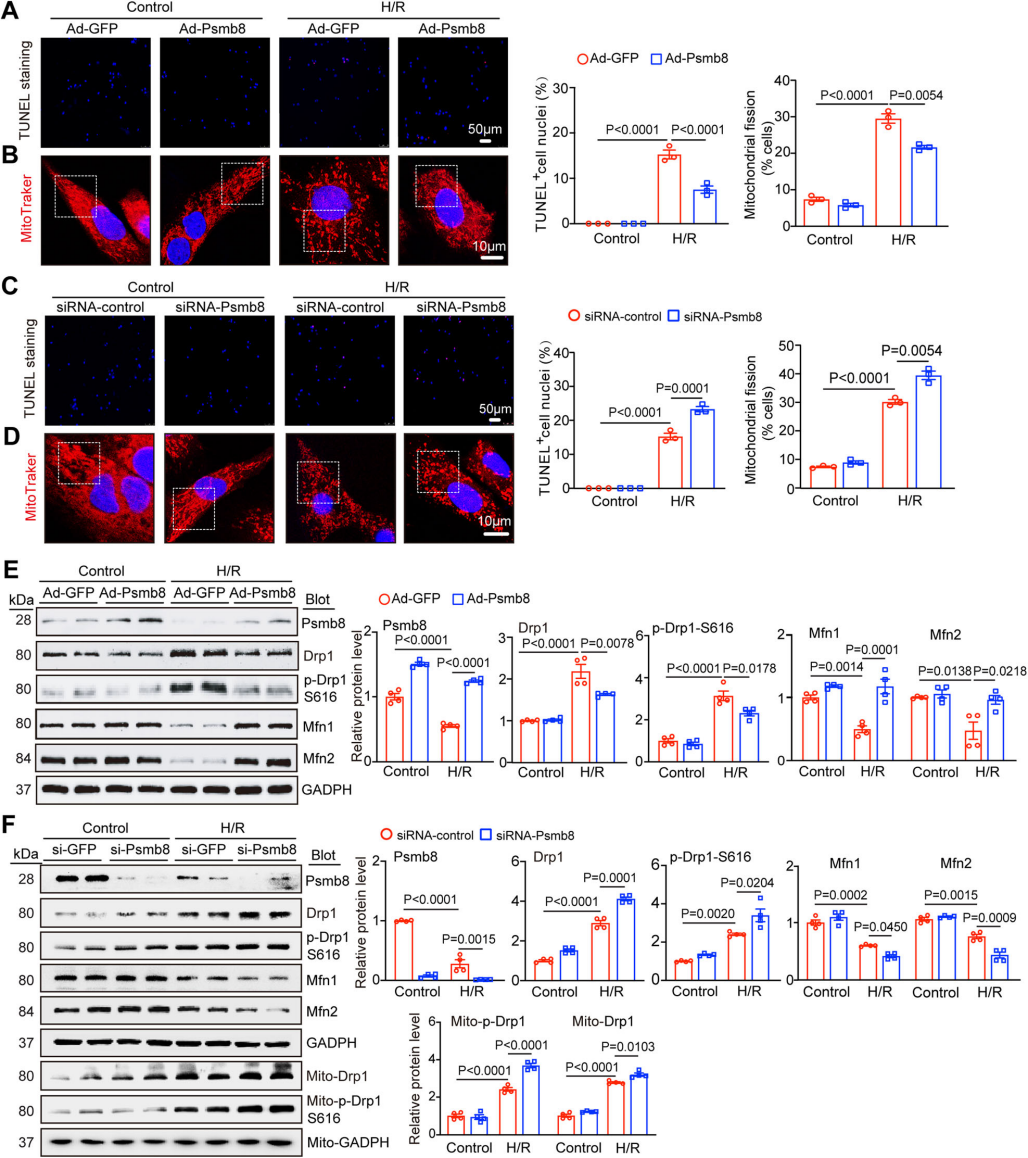
Overexpression of Psmb8 reduces H/R-stimulated cardiomyocyte apoptosis, ROS level and mitochondrial fission in vitro. **A** Neonatal rat cardiomyocytes (NRCMs) were infected with adenovirus expressing Ad-GFP or Ad-Psmb8 for 24 h and then simulated with H/R for another 24 h. Immunostaining of NRCMs with TUNEL (red) and DAPI (blue) to detect apoptotic cardiomyocytes (left). Quantification of the percentages of TUNEL-positive nuclei (right, *n* = 3 independent experiments). Bar: 50 μm. **B** Immunostaining of mitochondrial morphology was performed in cardiomyocytes using MitoTracker® Red (left). Quantification of fragmented mitochondria (right, *n* = 3 independent experiments). Bar: 10 μm. **C** NRCMs were infected with adenovirus expressing siRNA-control or siRNA-Psmb8 for 24 h and then stimulated with H/R for another 24 h. Immunostaining of NRCMs with TUNEL (red) and DAPI (blue) was performed to detect apoptotic cardiomyocytes (left). Quantification of the percentages of TUNEL-positive nuclei (right, *n* = 3 independent experiments). Bar: 50 μm. **D** Immunostaining of mitochondrial morphology in cardiomyocytes with MitoTracker® Red (left) and analysis of fragmented mitochondria (right, n = 3 independent experiments). Bar: 10 μm. **E** Detection of the Psmb8, Drp1, p-Drp1(S616), Mfn1, and Mfn2 protein levels with immunoblotting (left) and analysis of each protein level (right, *n* = 4 each group). **F** Detection of the Psmb8, Drp1, p-Drp1 (S616), Mfn1, Mfn2, Mito-Drp1 and Mito-Drp1 (S616) protein levels with immunoblotting (left) and analysis of each protein level (right, *n* = 4 each group). All values are expressed as the mean ± SEM, and n indicates the sample numbers per group.

Given that Psmb8 expression was specifically downregulated in H/R-induced NRCMs MI cardiomyocytes and colocalized with cardiomyocytes (Fig. 1D, E), we then examined the impact of Psmb8 on apoptosis and mitochondrial fission in neonatal rat cardiomyocytes (NRCMs) in vitro. NRCMs were first infected with an adenovirus expressing Psmb8 (Ad-Psmb8) and then exposed to H/R for 24 h. Green fluorescent protein (Ad-GFP) was used as a control. Compared with Ad-GFP infection, Ad-Psmb8 infection increased Psmb8 protein levels by approximately 1.5-fold (Fig. 5E) and dramatically suppressed the H/R-induced increases in the percentage of apoptotic cardiomyocytes (TUNEL assay) and mitochondrial division (MitoTracker red staining) (Fig. 5A, B). To validate these data, we performed Psmb8 knockdown experiments by infecting NRCMs with a scrambled control (siRNA-control) or small interfering RNA (siRNA) against Psmb8 (siRNA-Psmb8). As expected, knockdown of siRNA-Psmb8 markedly enhanced NRCM apoptosis and mitochondrial division compared with siRNA-control infection following 24 h of H/R exposure (Fig. 5C, D). However, overexpression or knockdown of Psmb8 did not affect cardiomyocytes under control condition (Fig. 5A–D).

To further assess whether Psmb8 regulates mitochondrial fission in vitro, we also analysed the protein levels of Drp1, Mfn1, and Mfn2 in NRCMs. Immunoblotting showed that, compared with Ad-GFP infection, Ad-Psmb8 overexpression substantially decreased the total Drp1 and phosphorylated (p)-Drp1 (S616) protein levels and increased the Mfn1 and Mfn2 protein levels following H/R stimulation (Fig. 5E). Conversely, knockdown of siRNA-Psmb8 had opposite effects (Fig. 5F). Moreover, the translocation of Drp1 to mitochondria was also checked through mitochondrial separation, and Drp1 and p-Drp1 protein levels were significantly increased in the siRNA-Psmb8 group (Fig. 5F). These data suggest that Psmb8 suppressed apoptosis and excessive mitochondrial fission in cardiomyocytes by improving Drp1-Mfn1/2 balance in vitro.

### Psmb8 binds to Drp1

**Fig. 6:**
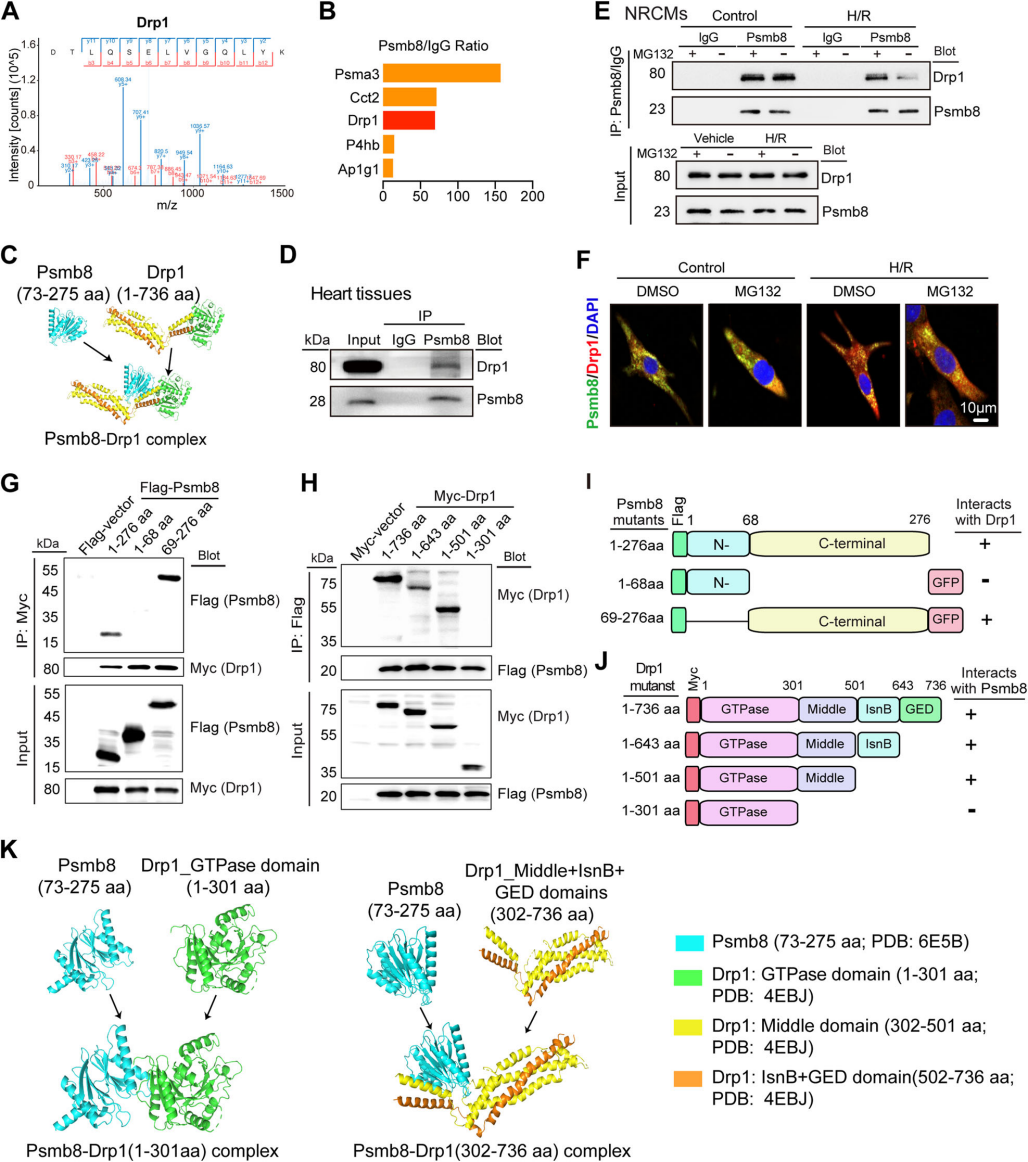
Psmb8 strongly associated with Drp1. **A** The MS/MS spectrum and extracted ion chromatograms from LC-MS/MS analysis of the derived Drp1 peptide. **B** Column chart showing the statistical proteins of LC-MS/MS analysis results. **C** The association between full-length Psmb8 and full-length Drp1 proteins was predicted by the ZDOCK server. Their structures were visualised using PyMOL software. **D** The association of endogenous Psmb8 with Drp1 protein in heart lysates, which were immunoprecipitated (IP) with IgG control or anti-Psmb8 antibody and then detected by immunoblotting analysis with anti-Drp1 or anti-Psmb8 antibody, respectively. **E** Analysis of the interaction between endogenous Psmb8 and Drp1 proteins in NRCM lysates treated with/without MG132 (20 μM) by IP with IgG control or anti-Drp1 antibody following by immunoblotting with antibody against Drp1 or Psmb8 under/not under H/R, respectively. **F** Fluorescence localisation of the Psmb8 and Drp1 proteins in NRCMs. **G** Identification of the Drp1-binding domains within the Psmb8. Whole cell lysates isolated from HEK293 cells co-transfected with Flag-tagged full-length Psmb8 or Flag-GFP-tagged Psmb8 mutants and Myc-tagged Drp1 plasmids were IP with anti-Myc antibody and analysed by immunoblotting with anti-Flag or Myc antibody. **H** Mapping of the Psmb8-binding domains within the Drp1. Whole cell lysates isolated from HEK293 cells co-transfected with Flag-tagged full-length Psmb8 and Myc-tagged full-length Drp1 or mutant plasmids were IP with anti-Flag antibody followed by immunoblotting with anti-Myc or Flag antibody. **I** Schematic diagram for the amino acids (69-276) of the Psmb8 binding to Drp1. **J** Schematic diagram for the amino acids (302-501) of Drp1 binding to Psmb8. **K** Prediction of the interaction between Psmb8 C-terminal fragment (73-275 aa) and the truncated Drp1 proteins (1-301aa, and 302-736 aa) by the ZDOCK server. The predicted structure was visualised by PyMOL software.

Given that Drp1 and Mfn1/2 are the central regulators of the balance between mitochondrial fission and fusion [20], and the stability of these proteins is modulated by ubiquitin E3 ligases and proteasome activity [21–24]. Interestingly, our data showed that the change in Drp1 but not Mfn1/2 protein expression was opposite to that in Psmb8 protein expression (Figs. 3 and 4). Furthermore, qPCR analysis indicated that Drp1 mRNA levels were similar between rAAV9-GFP-injected and rAAV9-Psmb8-injected mice and between WT and Psmb8-KO mice following sham or I/R injury, although an increase in Drp1 mRNA levels was observed in both groups following I/R stress (Fig. S4A, B), implying that Psmb8 reduces Drp1 protein levels through a posttranslational mechanism. To determine Drp1 is the direct target of Pmsb8 in cardiomyocytes, we first used liquid chromatography-tandem mass spectrometry (LC-MS/MS) to identify the protein substrates interacted with Psmb8 in NRCMs after the treatment of MG132, which prevents degradation of protein targets. The LC-MS/MS results showed that Drp1 interacted with Pmsb8, and ranked third based on the ratio of Psmb8/IgG (Fig. 6A, B). Therefore, we focused on assessing whether the Psmb8 influences Drp1 protein level through direct interaction in vivo and in vitro. We predicted whether the interaction between the Psmb8 and Drp1 proteins exits using the ZDOCK server (version 3.0.2), and the data revealed that the Psmb8 C-terminal fragment (73-275 aa) bound to full-length Drp1 (1-736 aa) proteins (Fig. 6C). Moreover, coimmunoprecipitation (Co-IP) analysis of heart tissues with an immunoglobulin G (IgG) control or an anti-Psmb8 antibody revealed that Drp1 proteins were markedly precipitated by the anti-Psmb8 antibody but not by the IgG control (Fig. 6D). Accordingly, we performed Co-IP assay antibody to test the interaction between endogenous Psmb8 and Drp1 in cultured NRCMs treated with or without MG132 (20 µM) under control or H/R condition. The results showed that the interaction level between Drp1 and Psmb8 was similar under control condition, whereas this interaction was reduced following H/R treatment, which was reversed by MG-132 treatment (Fig. 6E). Further, we conducted coimmunostaining with anti-Psmb8 and anti-Drp1 antibodies in NRCMs treated as in Fig. 6E, the results was consistent with Co-IP analysis (Fig. 6F). Thus, these findings suggest that Psmb8 might degraded through proteasome pathway.

To further confirm whether Psmb8 directly interacts with Drp1 and determine the binding sites between Psmb8 and Drp1, we transfected human embryonic kidney (HEK) 293 cells with Flag-tagged full-length Psmb8, Flag-GFP-tagged Psmb8 deletion mutants and Myc-tagged full-length Drp1. Co-IP experiments with an anti-Myc antibody showed that Flag-Psmb8 interacted with Myc-Drp1 and that amino acids 69 to 276 within the Psmb8 protein were essential for its binding to Drp1 (Fig. 6G, I). Furthermore, Myc-tagged full-length Drp1, various deletion mutants, and Flag-tagged full-length Psmb8 were transfected into HEK293 cells. Co-IP assays with an anti-Flag antibody revealed that Myc-Drp1 could also bind to Flag-Psmb8 but not to the empty vector control, and the middle domain (amino acids 302 to 501) within Drp1 was required for its binding to Psmb8 (Fig. 6H, J). Additionally, interactions between the Psmb8 C-terminal fragment (73-275 aa) and the truncated Drp1 protein, including the middle domain (302 to 501 aa), were confirmed by the ZDOCK server (Fig. 6K). Overall, these results indicate that Psmb8 is able to directly interact with Drp1 in cardiomyocytes.

### Psmb8 enhances Drp1 degradation

**Fig. 7:**
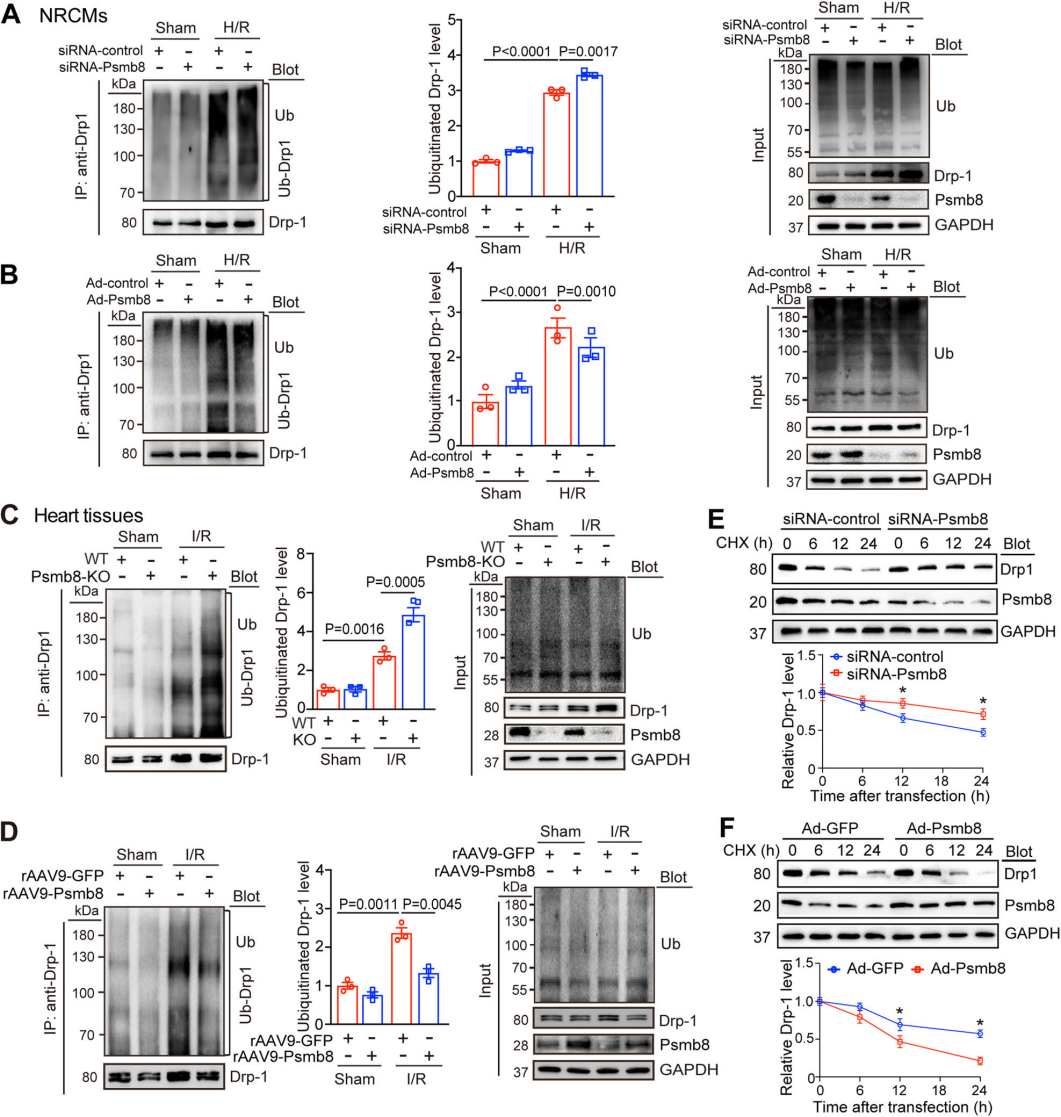
Psmb8 promotes Drp1 degradation. **A** Lysates from siRNA-control- or siRNA-Psmb8-infected NRCMs following H/R 24 h were IP with anti-Drp1 antibody and then analyzed with immunoblottong with anti-ubiquitin (Ub) or anti-Drp1 antibody (left). Input for each protein (right). Quantification of ubiquitinated Drp1 level (right, *n* = 3). **B** Lysates from NRCMs infected with Ad-GFP or Ad-Psmb8 and then following H/R 24 h were IP and then analyzed as described in (**A**). **C** WT and Psmb8-KO mice were subjected to sham or I/R surgery for 24 h. Heart lysates were immunoprecipitated with an anti-Drp1 antibody. Immunoblotting analysis of ubiquitin-conjugated Drp1 (left). Quantification of the relative ubiquitinated Drp1 protein level (middle, *n* = 3), and input of Psmb8 and Drp1 proteins (right). **D** rAAV9-Psmb8 or rAAV9-GFP-injected mice were subjected to sham or I/R surgery for 24 h. Heart lysates were immunoprecipitated and immunoblotting analysis of ubiquitin-conjugated Drp1 (left) as well as input (left) were performed as in (**C**). **E** NRCMs were first infected with adenovirus expression siRNA-control or siRNA-Psmb8 for 24 and then stimulated with CHX (10 μM) for another 6, 12 or 24 h. The protein levels of Drp1 and Psmb8 for each time point were determined by immunoblotting analysis with anti-Drp1 or anti-Psmb8 antibody (left) and quantification of the ubiquitinated Drp1 level (right; *n* = 3). **F** NRCMs were infected with Ad-GFP or Ad-Psmb8 and treated and analyzed as described in (**E**). All values are expressed as the mean ± SEM, and n indicates the number of independent experiments or samples per group.

Since Psmb8 has chymotrypsin-like protease activity that is responsible for the degradation of unnecessary proteins, we examined whether Psmb8 promoted the degradation of ubiquitinated Drp1 in NRCMs by an immunoprecipitation assay using an anti-Drp1 antibody. After 24 h of infection with siRNA-control or siRNA-Psmb8 and under the stimulation of H/R, immunoblotting with an antibody against ubiquitin (Ub) or Drp1 showed that knockdown of Psmb8 by siRNA-Psmb8 dramatically increased Drp1 ubiquitination and protein levels compared with those of the siRNA-control (Fig. 7A). In contrast, compared with Ad-GFP infection, Ad-Psmb8-mediated overexpression of Psmb8 reduced Drp1 ubiquitination and protein levels (Fig. 7B). To confirm the effect of Psmb8 on the stability of the ubiquitinated Drp1 protein in vivo, we performed an immunoprecipitation assay on heart tissues using an anti-Drp1 antibody. Immunoblotting revealed that Drp1 ubiquitination and protein levels were dramatically higher in lysates isolated from Psmb8-KO hearts than in those isolated from WT mice (Fig. 7C) but were markedly lower in the lysates of rAAV9-Psmb8-injected hearts than in those of rAAV9-GFP-injected hearts following I/R (Fig. 7D). Finally, we performed a cycloheximide (CHX) chase assay to examine whether Psmb8 affected the stability of the newly synthesised Drp1 protein in NRCMs. The results indicated that siRNA-Psmb8 infection markedly prolonged the half-life of the Drp1 protein compared with that in cells infected with siRNA-control (Fig. 7E); however, compared with Ad-GFP infection, Ad-Psmb8 infection reduced the half-life of the Drp1 protein (Fig. 7F). Thus, Drp1 is a newly identified substrate for Psmb8 in cardiomyocytes.

### Knockdown of Drp1 reverses I/R-mediated aggravation of myocardial injury in Psmb8-KO mice

**Fig. 8:**
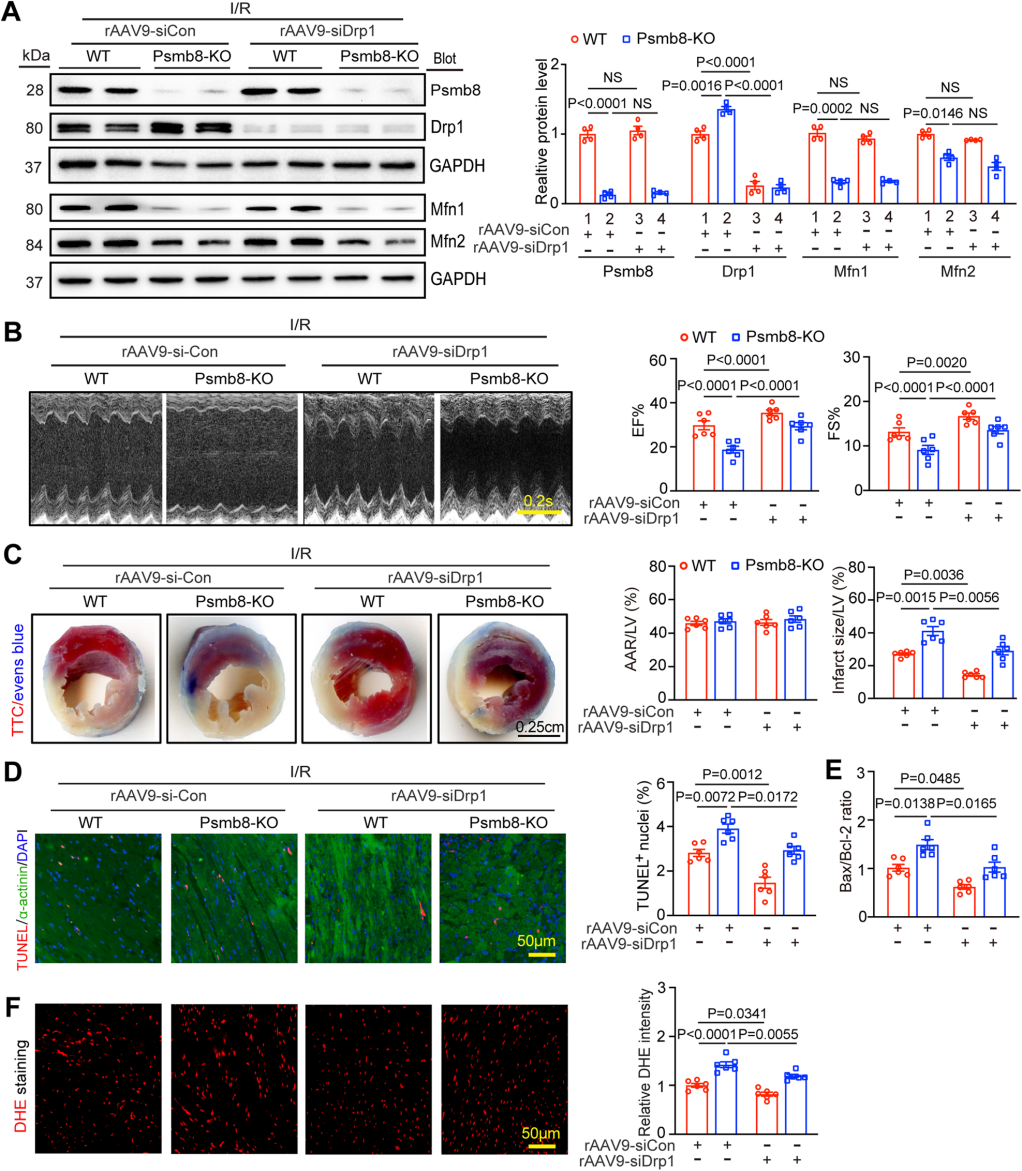
Knockdown of Drp1 in Psmb8-KO mice reverses I/R-induced cardiac damage and dysfunction. **A** Male WT or Psmb8-KO mice were injected with rAAV9-siCon or rAAV9-siDrp1 (1.0 × 10^12^ vg/mg) for three weeks and then exposed to I/R injury for another 24 h. The protein levels of Psmb8, Drp1, Mfn1 and Mfn2 were determined by immunoblotting (left), and the protein levels were quantified (right, *n* = 4). **B** Representative echocardiographic assessment of the function of the left ventricle (LV, left). The LV EF% and LV FS% were measured (left, *n* = 6). **C** The heart infarct area was examined by TTC and Evans blue staining (left). Bar: 2.5 mm. Analysis of the percentages of the area at risk (AAR) to the LV area and the infarct size to the LV area (right, *n* = 6). **D** Apoptotic cardiomyocytes in heart sections were evaluated by TUNEL (red), α-actinin (green) and DAPI (blue) staining (left). The percentages of TUNEL-positive nuclei are shown (right, *n* = 6). **E** The mRNA levels of Bax and Bcl-2 in the heart were determined by qPCR (*n* = 6). **F** Heart sections were stained with DHE dye (left), after which ROS concentrations were quantified (right, *n* = 6). Bar: 50 μm. The values are expressed as the mean ± SEM, and n indicates the number of samples per group.

To further evaluate whether Drp1 mediates the aggravative effects of Psmb8-KO on cardiac I/R injury in vivo, WT or Psmb8**-**KO mice were preinjected with rAAV9-siRNA-Drp1 (siDrp1) or rAAV9-siRNA-control (rAAV9-siCon) to knock down endogenous Drp1 in cardiac myocytes. After 3 weeks of delivery, all animals were then exposed to I/R injury for an additional 24 h. Immunoblotting indicated that Psmb8-KO or rAAV9-siDrp1 injection significantly downregulated Psmb8 and Drp1 protein levels in cardiac tissues compared with rAAV9-siCon injection (Fig. 8A). Meanwhile, Psmb8-KO decreased the protein levels of Mfn1 and Mfn2 in WT and Psmb8-KO mice (Fig. 8A, group 2 vs. 1; 4 vs. 3); however, knockdown of Drp1 by rAAV9-siDrp1 injection did not markedly change Mfn1 and Mfn2 protein levels compared with rAAV9-siConl-injected mice (Fig. 8A, group 3 vs. 1; 4 vs. 2). Consistent with the results in Fig. 4, the I/R-induced increases in cardiac dysfunction (reduced LV EF% and FS%), infarct area, the number of TUNEL^+^ myocytes, superoxide levels (DHE staining) and the ratio of Bax to Bcl2 observed in WT mice were all augmented in Psmb8**-**KO mice after injection of rAAV9-siCon (Fig. 8B–F, group 2 vs. 1, Table S5). However, these increases were greatly attenuated in Psmb8-KO mice with injection of rAAV9-siDrp1 (Fig. 8B–F, group 4 vs. 2). A similar protective role of rAAV9-siDrp1 injection in WT mice was also observed (Fig. 8B–F, group 3 vs. 1). Overall, these in vivo data indicate that Psmb8-KO promotes cardiac I/R injury possibly by increasing Drp1 activity.

## Discussion

**Fig. 9:**
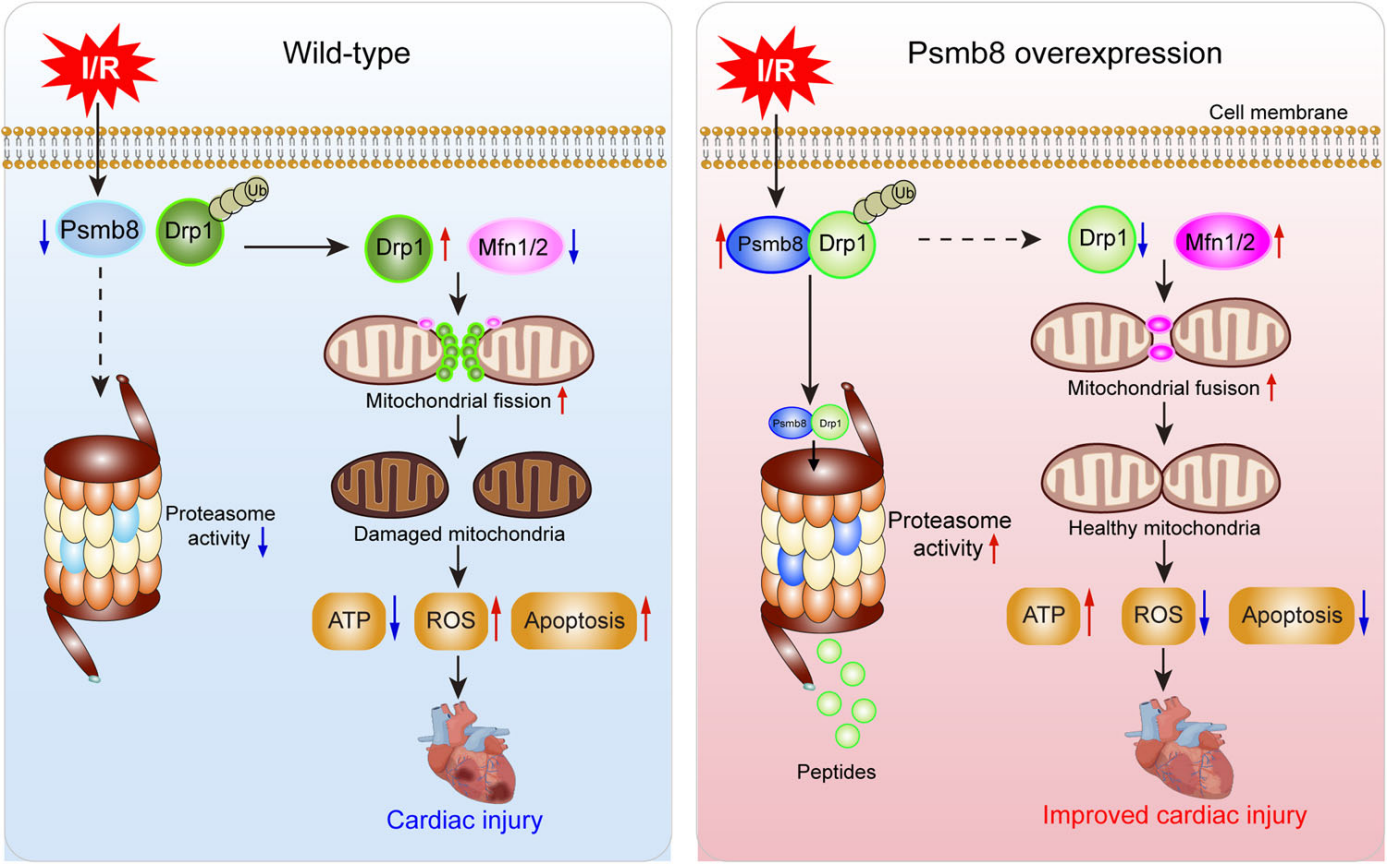
Schematic illustration. After I/R stress, Psmb8 expression in cardiomyocytes is downregulated, and causes a reduced interaction between Psmb8 and Drp1 proteins, which enhances Drp1 stability and subsequent excessive mitochondrial fission, leading to reduced ATP level and increased ROS production and apoptosis, all contributing to cardiac I/R injury. Conversely, overexpression of Psmb8 in cardiomyocytes increases Drp1 degradation and improves mitochondrial fission and fusion balance, thereby protecting heart against I/R injury.

The present study identified a novel role for Psmb8 in regulating cardiomyocyte mitochondrial homoeostasis during myocardial I/R injury in vivo and in vitro. Our results showed that Psmb8 overexpression in mice ameliorated I/R-induced cardiac dysfunction and injury associated with a reduction in mitochondrial fission by reducing Drp1 protein level. Conversely, Psmb8-KO in mice had the opposite effects. These observations were verified in cultured cardiomyocytes infected with Ad-Psmb8 or siRNA-Psmb8 in vitro. Mechanistically, Psmb8 directly associates with Drp1 and enhances its degradation, which limits excessive mitochondrial fission, ROS) production, ATP depletion, and subsequent cardiomyocyte apoptosis, thereby improving cardiac dysfunction. Therefore, our results suggest that Psmb8 is essential for maintaining cardiac mitochondrial dynamic balance by targeting Drp1, and highlight Psmb8 as a promising therapeutic target for cardiac I/R injury. A working model is presented in Fig. 9.

The immunoproteasome plays a central role in immunity and various inflammatory disorders. The expression levels of catalytic β immunosubunits, particularly β2i (Psmb10) and β5i (Psmb8), are dramatically upregulated in various tissues in mouse models in response to inflammatory and hypertensive stimuli and in human patients [5, 6]. Importantly, our recent studies indicate that the activation of Psmb10 and Psmb8 contributes to several cardiovascular diseases by regulating the stability of multiple protein substrates (ATG5, PTEN, ATRAP, IKBα, SOCS3, etc.) that are involved in autophagy, inflammation, oxidative stress, and apoptosis [7–12]. However, several studies have indicated that the expression levels and activities of the constitutive subunit (Psmb5) and immunosubunits (Psmb9 and Psmb10) are decreased in cardiac tissues and cells following ischaemic injury and are involved in cardiac and cerebral I/R injury [13, 17, 18]. For example, cardiomyocyte-specific overexpression of the β5-T60A mutation inhibits proteasome activity and dramatically exacerbates I/R-induced cardiac injury and dysfunction in mice, which is associated with increases in PTEN and PKC-δ protein levels and the inactivation of AKT [13]. Conversely, knockout of Psmb9 (β1i) abolishes ischaemic preconditioning-mediated cardioprotection against I/R injury by inhibiting PTEN degradation [14]. Furthermore, overexpression of PA28 (a proteasome activator) in cardiomyocytes prevents desmin-related cardiomyopathy and myocardial I/R injury [15]. More recently, we showed that cardiomyocyte overexpression of Psmb10 alleviated I/R-mediated cardiac infarction, apoptosis and dysfunction by targeting Parkin-Mfn1/2-mediated mitochondrial fusion [16]. However, the importance of another catalytic Psmb8 subunit in I/R-induced cardiac dysfunction has not been determined. Here, we extended the previous observations and further revealed that Psmb8 expression and activity were significantly reduced in mouse I/R-induced hearts and MI patients (Fig. 1). Overexpression of Psmb8 in cardiomyocytes significantly ameliorated I/R-induced cardiac dysfunction and damage, which were accompanied by decreased mitochondrial fission in mice (Figs. 2 and 3), and knockout of Psmb8 accelerated these effects (Fig. 4). The cardiomyocyte apoptosis and mitochondrial fission results in vivo were further confirmed in NRCMs infected with Ad-Psmb8 or siRNA-Psmb8 in vitro (Fig. 5). Thus, Psmb8 is involved in regulating mitochondrial fission and cardiac function during I/R injury.

The mechanisms responsible for the pathogenesis of cardiac I/R injury are complex. The disruption of mitochondrial homoeostasis is a central factor in the regulation of cardiac I/R damage [3, 25, 26]. Notably, Drp1 is a key fission protein that hydrolyses GTP to trigger mitochondrial fission, and this effect is facilitated by numerous adapter proteins, such as fission protein 1 (Fis1), mitochondrial fission factor (MFF), and mitochondrial dynamics proteins of 49 kDa and 51 kDa (MID49 and MID51, respectively), at the outer mitochondrial membrane (OMM) [20]. Moreover, Drp1-mediated mitochondrial fission is a major source of ROS, which play a pivotal role in cardiomyocyte death during I/R injury [3, 4]. Conversely, inhibition of Drp1 markedly reduces mitochondrial fission and apoptosis [20]. Thus, selective targeting of Drp1 may be a new therapeutic strategy for ischaemic disease. Multiple posttranslational modifications, including phosphorylation, SUMOylation, and ubiquitination, regulate Drp1 activity, translocation, and degradation [25]. Pathophysiological stimuli, such as ATP depletion, hypoxia and I/R injury, can trigger the translocation of Drp1 to the OMM through the phosphorylation/dephosphorylation of serine residues 616 and 637 [3]. Currently, several protein kinases, including CDK1, PKCδ, CaMKII, and PINK1, can phosphorylate Drp1 at serine 616 (Drp1^S616^), which leads to increased activity and subsequent mitochondrial fission [3, 27], while the Ca^2+^-dependent phosphatase calcineurin catalyses Drp1 dephosphorylation at serine 637 to inhibit mitochondrial fission, thereby ameliorating I/R-induced cardiac cell death and dysfunction [28–30]. Moreover, the stability of Drp1 is modulated by E3 ligase-dependent ubiquitination. Mitochondrial MARCH5 (MITOL) can promote Drp1 ubiquitination and proteasomal degradation and limit mitochondrial fission [21–23]. The E3 ligase Parkin also mediates Drp1 ubiquitination and degradation, resulting in an imbalance in mitochondrial dynamics in Parkinson’s disease [24], suggesting that ubiquitin modification regulates Drp1 stability and mitochondrial fission. Notably, Parkin protein levels are increased in I/R-induced hearts [16], which is consistent with the pattern of Drp1 expression observed in the present study (Figs. 3G, 4H), and exclude the involvement of Parkin in Drp1 degradation. Interestingly, our recent studies revealed that the I/R-induced upregulation of Drp1 expression is markedly reduced by several small molecule components, such as ursolic acid (UA), MK-886 and TCH-165, by increasing the expression and activity of immunosubunits (Psmb9, Psmb10 or Psmb8) [17, 18, 31]. However, the mechanism by which these immunosubunits enhance ubiquitinated Drp1 degradation has not been determined. Here, our data revealed a new mechanism by which Psmb8 directly interacted with Drp1 and enhanced its degradation (Figs. 6, 7). Moreover, knockdown of Drp1 by treatment with si-Drp1 in Psmb8-KO mice robustly attenuated I/R-induced cardiomyocyte apoptosis and mitochondrial fission (Fig. 8), suggesting that Drp1 is a direct target of Psmb8 in cardiomyocytes. However, Psmb8-KO did not affect the stability of Drp1 receptor proteins such as MFF, MID49 and MID51 (Fig. S2) Thus, we identified a novel mechanism by which Psmb8 ameliorates cardiac I/R injury and dysfunction through improving the balance of mitochondrial dynamics by targeting Drp1 degradation.

To date, accumulating evidence has demonstrated that proteasomal activation is involved in different cardiovascular diseases induced by hypertension, angiotensin II infusion and pressure overload [7–11]. In contrast, deletion or inhibition of proteasome catalytic subunits (Psmb9, Psmb10 or Psmb5) and activators (PA28) are associated with exacerbation of cardiac I/R injury and cardiomyopathy [14, 15, 17, 18], suggesting that these factors could be novel targets for therapeutic intervention in hypertrophic or ischaemic heart diseases. The inhibition of proteasome activity with inhibitors such as bortezomib, epoxomicin, and PR-957 (ONX-0914) significantly attenuates hypertension, cardiac remodelling, heart failure, atrial fibrillation and retinopathy after angiotensin II infusion or pressure overload. [7–11, 32, 33] More recently, we identified several immunoproteasome inducers, including ursolic acid (UA), MK-886, TCH-165, and N-arachidonoylphenolamine (AM404) [34], which can enhanced the expression levels and activities of immunosubunits (Psmb10, Psmb9 and Psmb8) and target the PP2A-AMPK, keap1-NRF2 and Drp1 signalling pathways, thereby protecting against cardiac I/R damage [17, 18, 31].

This study has several limitations. First, we did not determine the mechanisms by which I/R downregulates Psmb8 expression in cardiomyocytes; which specific E3 ligases promote Drp1 ubiquitination for degradation by Psmb8; how Psmb8 increases Mfn1 and Mfn2 expression in cardiomyocytes, or whether the activation of Psmb8 represents a therapeutic strategy for ischaemic heart disease in human patients. Moreover, our patient sample size was relatively small, and further large-scale multicentre studies are needed to verify our findings.

In summary, the present study revealed that Psmb8 expression was significantly decreased in I/R-induced hearts and H/R-induced cardiomyocytes, as was that in patients with MI. An increase in Psmb8 expression significantly attenuated I/R-mediated cardiac injury and dysfunction by targeting Drp1 for degradation and inhibiting excessive mitochondrial fission. Taken together, these findings provide new insights into the mechanism by which Psmb8 regulates Drp1 stability and subsequent cardiac I/R injury and suggest that activating Psmb8 may be a promising therapeutic strategy for ischaemic heart disease.

## Materials and methods

### Animals

C57BL/6J WT and Psmb8-KO (stock Psmb8tm1Hif/J) mice were obtained from the Jackson Laboratory (Bar Harbour, USA) as previously reported [7]. The animals were kept in standard cages at 24–25 °C and received ad libitum access to standard mouse chow and tap water. The experimental procedures were approved by the Ethics Committee of Animal Experiments at Chao-Yang Hospital (2020-Animal-164) and conformed to the Guidelines for the Care and Use of Laboratory Animals issued by the National Institutes of Health (1996, USA).

### Construction of recombinant adeno-associated virus type 9 (rAAV9)

Recombinant adeno-associated virus type 9 (rAAV9) carrying GFP (rAAV9-GFP), Psmb8 (rAAV9-Psmb8) or short hairpin RNA (shRNA) to knock down Drp1 (rAAV9-si-Drp1) or a control shRNA (rAAV9-siCon) were produced by Shanghai HanBio Biotechnology (China) as previously described [7]. Male wild-type mice were intravenously injected with rAAV9-GFP or rAAV9-Psmb8 (1.8 × 10^12^ vg/mL). The primer sequences to knock down endogenous Drp1 in cardiomyocytes were as follows: forward, 5’-CGGTGGTGCTAGGATTTGTTA-3’; reverse, 5’-TAACAAATCCTAGCACCACCG-3’. For the Drp1 knockdown experiment, male WT or Psmb8-KO mice were intravenously injected with rAVV9-siCon or rAAV-siDrp1 (1 × 10^12^ vg/mg). After 3 weeks, the animals were subjected to sham or I/R surgery. Twenty-four hours later, the hearts were harvested for further analysis of ischaemic injury.

### Generation of the cardiac I/R mouse model and drug treatments

Male WT mice (8–10 weeks old) were randomly divided into four groups: sham+rAAV9-GFP, sham+rAAV9-Psmb8, I/R + rAAV9-GFP, and I/R + rAAV9-Psmb8 (*n* = 6 per group); WT or Psmb8-KO mice were randomly divided into four groups: sham+WT, sham+Psmb8-KO, I/R + WT and I/R+Psmb8-KO (*n* = 6 per group). Mice were anaesthetised by isoflurane inhalation (1.5–2%). The establishment of myocardial I/R in mice was performed by occlusion of the left anterior descending artery (LAD) to induce ischaemia for 30 min followed by reperfusion for 6 or 24 h, as previously described [35]. To measure the infarct area, the mice (*n* = 6 per group) were intraperitoneally injected with an overdose of 2,2,2-tribromoethanol (8 mg/kg). After ligation of the LAD, approximately 900 μl of 1% Evans blue dye was injected into the left ventricle (LV). The hearts were quickly frozen, cut and then incubated with 1% 2,3,5-triphenyltetrazolium chloride (TTC) solution for 20 min. Four slices of each heart were analysed with Image-Pro Plus software. The infarct size, area at risk (AAR), and LV area of each section were analysed with Image-Pro Plus software. The percentages of the infarct area/LV area and (infarct area+ area at risk)/LV area were calculated as previously described [16–18]. The mice were sacrificed by an overdose of tribromoethanol after I/R. Heart tissues and blood samples were harvested for further experiments.

### Echocardiographic measurements

The mice (*n* = 6 per group) were anaesthetised via inhalation of 2% isoflurane. Echocardiography was performed using a Vevo 2100 system (MS-400, VisualSonics, Canada). The parameters for the LV internal dimension (LVID), LV posterior wall (LVPW), LV anterior wall (LVAW), LV volume (LV Vol), ejection fraction (EF%) and fractional shortening (FS%) were calculated as previously reported [16].

### Histological examination

The hearts of WT, rAAV9-injected and KO mice (*n* = 6/group) were routinely fixed in 10% formalin solution and then embedded in paraffin. Apoptosis in heart sections (4 µm thick) or neonatal rat cardiomyocytes (NRCMs) was analysed using a TUNEL fluorescence FITC kit (Roche, USA) according to the manufacturer’s protocols. The myocardia and nuclei were identified by staining with an antibody against a-actinin (green) and DAPI (blue, Sigma‒Aldrich), respectively. The hearts were embedded in OCT solution (Sakura, 4583), cut into 4 mm-thick slices, and incubated with 1 µM DHE in PBS for 30 min to examine total ROS levels. Psmb8 expression in cardiomyocytes was examined by staining with antibodies against Psmb8 (1:200, 13726) or ɑ-actinin (1:400, 11313-2-AP). Fluorescent images were acquired using a Leica DM2500. Five to six random fields were obtained from each slide, and the positive areas were quantified using NIH ImageJ software (NIH) [16–18].

### Liquid chromatography coupled with tandem mass spectrometry analysis (LC–MS/MS) and bioinformatics analysis

Male wild-type mice were injected with rAAV9-GFP and rAAV9-Psmb8 and subjected to sham surgery or I/R. After 24 h, the mice were euthanized by an intraperitoneal injection of an overdose of 150 mg/kg pentobarbital sodium. Five heart samples from each group were homogenised in lysis buffer, and the lysates were digested with trypsin (Promega) and labelled with different tandem mass tag/iTRAQ kit reagents (Thermo Fisher Scientific) based on the manufacturer’s instructions. The dried peptides were analysed by liquid chromatography coupled with tandem mass spectrometry (LC–MS/MS) as previously described [36]. The MS proteomics data are shown in Table S3. A heatmap of the differentially expressed proteins (DEPs) was generated using R version 4.3.1 software. Gene Ontology (GO) enrichment and WIKI pathway analyses were used to identify enriched proteins associated with cardiac I/R injury in the dataset as previously described [36].

### Transmission electron microscopy (TEM)

Heart samples were isolated from mice subjected to I/R and sham surgery (*n* = 3–4/group). Ischaemic LV tissues were cut into approximately 1 mm^3^ blocks and incubated in 2.5% glutaraldehyde at 4 °C for 24 h. Then, the fixed blocks were placed in 1% osmium tetroxide (OsO_4_) for 2 h, dehydrated, and embedded in epoxy resin. The blocks were sliced into 50–70 nm-thick sections with an ultramicrotome. The slides were double-stained with uranyl acetate for 30 min and then with lead citrate for 2 min and finally examined at a magnification of ×15,000 with an HT7700 transmission electron microscope. Approximately 100 mitochondria in each field were photographed in five to six random fields in each slide. The mitochondria were analysed using Image-Pro Plus software. Mitochondria with areas <0.6 μm^2^ were defined as fragmented [16, 30].

### Analysis of ATP levels

Heart tissues were homogenised in lysis buffer on ice, and ATP levels in the supernatants (20 μl) were determined by adding 100 μl of ATP detection working solution according to the manufacturer’s protocols (#S0027, Beyotime). The fluorescence intensity was measured with a microplate reader.

### Measurement of proteasome activity in the hearts and serum

Proteasome activity in mouse heart tissues or human serum samples was measured using fluorogenic peptides as previously reported [16, 17]. Heart tissue lysates (20 μg) or serum (25 μl) were added to 100 μl of reaction buffer containing fluorogenic substrates in the presence of 20 μmol/L MG-132 or 5 μmol/L epoxomicin. The mixture was subsequently incubated at 37 °C for 10 min. The following fluorogenic peptides were used: Z-LLE-AMC (45 μmol/L, caspase-like), Ac-RLR-AMC (40 μmol/L, trypsin-like) and Suc-LLVY-AMC (18 μmol/L, chymotrypsin-like). The fluorescence intensities were determined at excitation and emission wavelengths of 380 nm and 460 nm, respectively.

### Quantitative real-time PCR analysis

Total RNA was isolated from fresh heart tissues or from NRCMs with TRIzol reagent (Invitrogen) according to the manufacturer’s protocols. First-strand cDNA was generated by reverse transcription of total RNA (1–2 μg). The expression levels of each gene, including Psmb6, Psmb7, Psmb5, Psmb9, Psmb10, Psmb8, Drp1, Mfn1, and Mfn2, were measured on a PCR thermocycler (S1000 Thermal Cycler, USA) using specific primers (Table S5). The values obtained were normalised to the level of the GAPDH gene [16].

### MtDNA copy number

NADH dehydrogenase subunit 1 (ND-1) was analysed by qPCR to determine changes in mitochondrial DNA copy numbers in the heart. The β-globin gene was used as the control. The primer sequences are listed in Table S5.

### Extraction of cell mitochondria and Immunoblotting analysis

The mitochondrial protein of NRCMs was extracted by cell mitochondrial protein separation kit (C3601, Beyotime) according to the manufacture’s instructions. Commercial radioimmunoprecipitation assay (RIPA) lysis buffer containing a protease inhibitor cocktail (1:100) was used to lyse primary cardiomyocytes, primary fibroblasts, and myocardial tissue. Equal amounts of protein (40–50 μg) were separated by 8–10% SDS‒PAGE and then transferred to a PVDF membrane, which was incubated with the primary antibody overnight. The signal intensity of each blot was measured and standardised to the GAPDH expression level with a Gel-Pro 4.5 analyser (Media, USA) as previously described [16]. Primary antibodies were obtained from Cell Signalling Technology Corp. (Devers, USA): Psmb9 (1:1000, 87667S), Psmb10 (1:1000, 17579S), Psmb8 (1:1000, 13635S), Drp1 (1:2000, 5391S), phospho-Drp-1-Ser616 (1:1000, 3455S), and GAPDH (1:5000, 5174S). Bax (1:1000, 50599-2-IG), Bcl-2 (1:1000, 26593-1-AP), ubiquitin (Ub, 1:1000, 10201-2-AP), Mfn1 (1:2000, 13798-1-AP), and Mfn2 (1:2000, 12186-1-AP) were obtained from Proteintech Group, Inc. (Chicago, USA).

### Immunoprecipitation and Ubiquitilation analysis

Immunoprecipitation was performed as previously reported [7, 16]. Heart tissues or NRCMs were lysed using lysis buffer containing 20 mM TrisHCl (pH 7.5), 150 mM NaCl, 0.1% SDS, and 1% NP-40 with protease inhibitor cocktail (Roche). For Drp1 ubiquitylation assay, heart tissues or NRCMs were lysed using lysis buffer containing 50 mM Tris-HCl (pH 7.4), 150 mM NaCl, 1% Triton X-100, 0.1% SDS, and 0.5% sodium deoxycholate, with protease inhibitor cocktail (Roche) [37]. HEK293T cells were transfected with Flag-tagged full-length Psmb8 and Myc-Drp1, as well as their mutants, for 24 h and then lysed in lysis buffer on ice for 20 min. The lysates were centrifuged, and the protein levels were quantitated with a bicinchoninic acid (BCA) kit (Thermo Fisher Scientific). One milligram of cell extract was mixed with 2 μg of primary antibody and 50 μl of protein A/G magnetic beads (#88803; Thermo Scientific Pierce) and incubated at 4 °C overnight. After centrifugation, the pellets were washed twice with wash buffer. The bound proteins were boiled in 2× loading buffer and then separated by 8 or 10% SDS‒PAGE followed by immunoblot analysis with antibodies against Psmb8, Drp1, Myc, or Flag. The protein bands were analysed with a Gel-Pro 4.5 analyser or NIH ImageJ [7].

### Mass spectrometry (MS)

Following treatment with MG132 (20 µM) in NRCMs, IP was performed using an anti-Psmb8 primary antibody. Precipitates were separated using SDS-PAGE gels and were subsequently stained with silver nitrate. Bands that differed in the IP-Psmb8, IgG, and input groups were analyzed using liquid chromatograph-electrospray ionisation-mass spectrometry (Jingjie Biotechnology, Hangzhou, China).

### Prediction of the protein interaction between Psmb8 and Drp1

The interaction between Psmb8 C-terminal fragment (73-275 aa, PDB code: 6E5B, chain Y) and The interaction between the Psmb8 C-terminal fragment (73-275 aa, PDB code: 6E5B, chain Y) and the full-length Drp1 protein, as well as the truncated Drp1 protein (1-301aa and 302-736 aa, PDB code: 4EBJ), was predicted by the ZDOCK server. The predicted structure was visualised by PyMOL software (version 2.5.7). The selected PDB files have some missing residues. Specifically, in 6e5b.pdb, the N-terminal tail domain of Psmb8 is not in the crystal set. The 20S proteasome subunit Psmb8 was originally expressed as a precursor with 276 amino acids. After complex assembly, the 72 amino acids in the peptide N-terminal fragment of the Psmb8 subunit are cleaved, forming the mature Psmb8 subunit of the 20S complex [38]. Therefore, we did not incorporate these residues in our simulations.

### Neonatal rat cardiomyocytes isolation and culture

Neonatal rat cardiomyocytes (NRCMs) were isolated from the heart tissues of 1-day-old Sprague–Dawley rats with 0.07% type II collagenase (Sigma–Aldrich) as previously reported [7]. The cells were incubated in fresh culture medium (DMEM/F12 containing 15% FBS at 37 °C and 5% CO_2_ for 1.5 h to separate cardiac fibroblasts from cardiomyocytes. After that, the isolated cell supernatant was centrifuged at 23 °C for 5 min and then re-suspended in culture medium (DMEM/F12 with 10% FBS) for 24 h.

### Recombinant adenovirus infection and hypoxia/reoxygenation (H/R) of NRCMs

Recombinant adenoviruses vectors expressing Psmb8 (Ad-Psmb8), GFP alone (Ad-GFP), small interfering RNA against Psmb8 (siRNA-Psmb8), Drp1 (siRNA-Drp1) or scramble siRNA-control (siRNA-control) were produced with the pAdEasy system by Shanghai HanBio Biotechnology (China) as previously described [7]. NRCMs were infected with these adenoviral vectors at a multiplicity of infection (MOI) of 50 for 24 h and then exposed to hypoxia/reoxygenation (H/R) for an additional 24 h as previously reported [7, 39]. To induce H/R in vitro, NRCMs were cultured with simulated ischaemia buffer containing NaHCO_3_, NaCl, KCl, NaH_2_PO_4_·2H_2_O, anhydrous CaCl_2_, MgCl_2_·6H_2_O, sodium lactate, 2-D-ribose, and 2-deoxyglucose and incubated at 37 °C and 1% O_2_ (hypoxic conditions) for 6 h. Then, the cells were transferred to normal cell medium (reoxygenation) and cultured under normal conditions for 24 h as previously reported [17].

### Mitochondrial and superoxide staining

Mitochondrial morphology in cardiomyocytes was examined as previously reported [16, 17, 30]. Briefly, NRCMs were cultured on coverslips coated with 0.01% poly-L-lysine in DMEM for 24 h. The medium was removed, and staining solution containing the mitochondrion-selective probe MitoTracker Red CMXRos (0.02 µM, Molecular Probes) was added and incubated for 20 min. The stained mitochondria were imaged with a Zeiss confocal microscope (LSM510 META). Five or six visual fields were randomly analysed in each group to determine mitochondrial fission (*n* = 3 independent experiments). Image Pro Plus was used to analyse the mitochondrial fission level, and a punctiform mitochondrial phenotype was scored when at least 90% of the tubular mitochondria were disintegrated [40]. To examine superoxide production, heart sections (4 µm thick) or NRCMs were stained with DHE (1 μM in PBS) as previously reported [16, 17]. The images were visualised using a fluorescence microscope with excitation and emission wavelengths of 488 nm and 560 nm, respectively. Five or six visual fields from each sample were obtained using a Leica DM2500, and the DHE-positive areas were analysed with NIH ImageJ software.

### Colocalization of Psmb8 and Drp1 in cardiomyocytes

NRCMs were stained with antibodies against Psmb8 and Drp1. Fluorescence images were acquired using a Leica DM2500. and analysed using NIH ImageJ software.

### Pulse-chase assay

NRCMs were infected with Ad-GFP alone, Ad-Psmb8, siRNA-control, or siRNA-Psmb8 for 24 h. Cell lysates were harvested at 6, 12, or 24 h after treatment with 10 μM cycloheximide (CHX). Endogenous Drp1 and Psmb8 protein levels were measured by immunoblot analysis with anti-Drp1 and anti-Psmb8 antibodies, respectively, and then normalised to the levels of GAPDH as previously described [7].

### Study of human patients

The Psmb8 expression levels in normal controls and patients with MI were analyzed based on a published single-cell transcriptome database as reported previously [19]. For our analysis, we used the cell phenotypes and cell coordinate information provided by the original study. Using the FindMarkers function in Seurat (version 5.1.0), differential expression of the Psmb8 gene between the control group and MI patients was identified in both cardiac tissue and cardiomyocyte cluster. The adjusted P-values and fold change values obtained from the FindMarkers analysis were used to create bar plots.

### Statistical analysis

The data are expressed as the means ± standard errors of the means (SEMs), and Prism version 9.4.1 and R version 4.3.1 were used for all the statistical examinations. For normally distributed values with equal variances, the differences between two groups were analysed with Student’s t test. Differences among more than two groups were analysed with one-way or two-way ANOVA followed by Bonferroni’s post hoc correction. The nonparametric Mann‒Whitney U test was used. Otherwise, the statistical significance of the data with uneven variances was evaluated using the Kruskal‒Wallis test. *P* < 0.05 were considered to indicate statistical significance. A paired t test was used to analyse patient data. The relationship between serum Psmb8 concentrations or chymotrypsin-like activity and the incidence of STEMI was analysed using conditional logistic regression models. *P* < 0.05 indicated a statistically significant difference.

## Supplementary information

Supplementary data

Supplementary Table 2

Supplementary Data_MS proteins

Supplementary Data-WB original figures

## Data Availability

All data needed to evaluate the conclusions in the paper are present in the paper and/or the Supplementary Materials.
