# Correction: Psmb8 inhibits mitochondrial fission and alleviates myocardial ischaemia/reperfusion injury by targeting Drp1 degradation

**DOI:** 10.1038/s41419-025-07408-3

**Published:** 2025-02-14

**Authors:** Hui-Xiang Su, Luo-Luo Xu, Pang-Bo Li, Hai-Lian Bi, Wen-Xi Jiang, Hui-Hua Li

**Affiliations:** 1 Department of Emergency Medicine, Beijing Key Laboratory of Cardiopulmonary Cerebral Resuscitation, Beijing Chaoyang Hospital, Capital Medical University, Beijing, China; 2 Institute of Cardiovascular Diseases, First Affiliated Hospital of Dalian Medical University, No.193, Lianhe Road, Xigang District, Dalian, China

**Keywords:** Myocardial infarction, Mechanisms of disease

Correction to: *Cell Death and Disease* 10.1038/s41419-024-07189-1, published online 08 November 2024

The authors noticed that the images of the TTC/Evans blue staining in the sham+WT group in Fig. 4B and the DHE staining in sham+WT, sham+Psmb8-KO and WT+I/R groups in Fig. 4C were shown incorrectly. The correct Fig. 4B and 4C are shown below. The corrections do not affect the conclusions of the study. The authors would like to apologize for any inconvenience caused.
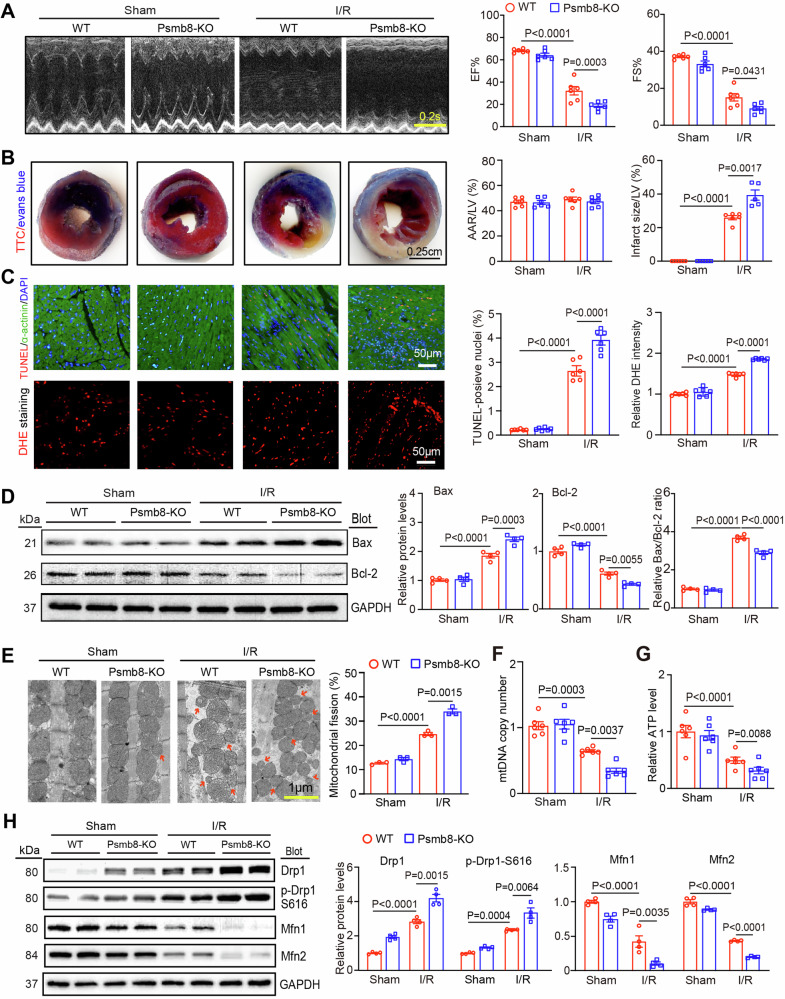


The original article has been corrected.

