# Supplementary material for: Psmb8 inhibits mitochondrial fission and alleviates myocardial ischaemia/reperfusion injury by targeting Drp1 degradation
Source: Cell Death Dis. 2024 Nov 8;15(11):803. doi: 10.1038/s41419-024-07189-1 (PMC11549449; doi:10.1038/s41419-024-07189-1)
Supplement: Supplementary file 1 — Supplementary data [file 41419_2024_7189_MOESM1_ESM.docx]

**Supplementary Materials for**

**Pmsb8 inhibits mitochondrial fission and alleviates myocardial ischaemia/reperfusion injury by targeting Drp1 degradation**

Hui-Xiang Su^1#^, Luo-Luo Xu^1#^, Pang-Bo Li^1^, Hai-Lian Bi^2^, Wen-Xi Jiang^1^* and Hui-Hua Li^1^*

^1^Department of Emergency Medicine, Beijing Key Laboratory of Cardiopulmonary Cerebral Resuscitation, Beijing Chaoyang Hospital, Capital Medical University, Beijing 100020, China

^2^Institute of Cardiovascular Diseases, First Affiliated Hospital of Dalian Medical University, No.193, Lianhe Road, Xigang District, Dalian 116011, China

Corresponding authors. Huihua Li ([hhli1935@aliyun.com](mailto:hhli1935@aliyun.com)) or Wenxi Jiang (wenxi_jiang@163.com)

^#^These authors contributed equally

**Table S1.** Echocardiographic measurement of wild type (WT) mice with rAAV9-GFP and rAAV9-Pmsb8 subjected to Ischemia-Reperfusion for 24 hours.

| Parameter | rAAV9-GFP  Sham | rAAV9-Pmsb8  Sham | rAAV9-GFP  I/R | rAAV9-Pmsb8  I/R |
| --- | --- | --- | --- | --- |
| n | 6 | 6 | 6 | 6 |
| HR (bpm) | 519.9±7.834 | 514.6±9.715 | 489.5±6.682* | 496.3±8.419 |
| EF (%) | 65.88±2.425 | 62.69±2.151 | 27.66±2.075** | 39.15±2.312^##^ |
| FS (%) | 35.46±1.795 | 33.39±1.564 | 13.28±1.162** | 18.92±1.346^##^ |
| LV Mass (AW)(mg) | 111.0±3.063 | 121.1±7.120 | 120.3±10.16 | 109.6±6.632 |
| LVID; d (mm) | 3.532±0.089 | 3.764±0.089 | 4.337±0.092** | 3.913±0.094^##^ |
| LVID; s (mm) | 2.264±0.117 | 2.522±0.114 | 3.767±0.116** | 3.196±0.114^##^ |
| LVPW; d (mm) | 0.8877±0.040 | 0.9560±0.051 | 0.7159±0.076 | 0.7212±0.053 |
| LVPW; s (mm) | 1.260±0.067 | 1.362±0.082 | 0.9107±0.089* | 0.9190±0.087 |
| LVAW; d (mm) | 0.8898±0.076 | 0.7946±0.038 | 0.7448±0.064 | 0.8383±0.078 |
| LVAW; s (mm) | 1.422±0.063 | 1.293±0.028 | 0.8702±0.093** | 1.098±0.105 |
| LV Vol; d (μl) | 52.37±3.244 | 58.58±4.618 | 85.15±4.183** | 71.56±2.387^#^ |
| LV Vol; s (μl) | 18.00±2.424 | 28.21±4.860 | 61.30±4.308** | 43.89±2.525^##^ |

Values are mean ± SEM, n = 6 per group. HR, heart rate; EF, ejection fraction; FS, fraction shortening; LV Mass, left ventricular mass; LVID, left ventricular internal diameter; LVPW, left ventricular posterior wall; LVAW, left ventricular anterior wall; LV Vol, left ventricular volume. **P* < 0.05, ***P* < 0.01 versus rAAV9-GFP+sham; ^#^*P* < 0.05, ^##^*P* < 0.01 versus WT+I/R.

**Table S2.** This table is an independent Excel file which contains a lot of data. If necessary, we will upload it separately.

**Table S3.** Echocardiographic measurement of wild type (WT) and Pmsb8-KO mice subjected to I/R for 24 hours.

| Parameter | WT+Sham | Pmsb8-KO+sham | WT+I/R | Pmsb8-KO+I/R |
| --- | --- | --- | --- | --- |
| n | 6 | 6 | 6 | 6 |
| HR (bpm) | 531.5±2.784 | 528.5±3.566 | 490.1±5.932** | 489.8±6.969 |
| EF (%) | 67.72±0.973 | 62.85±1.576 | 28.06±3.957** | 17.07±1.561^#^ |
| FS (%) | 36.91±0.480 | 32.50±1.253 | 13.13±1.978** | 8.164±0.966^#^ |
| LV Mass (AW; mg) | 90.86±3.847 | 87.21±3.953 | 97.68±3.344 | 88.38±9.297 |
| LVID; d (mm) | 3.373±0.093 | 3.621±0.125 | 3.948±0.098** | 4.254±0.082^#^ |
| LVID; s (mm) | 2.088±0.084 | 2.449±0.117 | 3.445±0.119** | 3.946±0.076^##^ |
| LVPW; d (mm) | 0.788±0.034 | 0.702±0.038 | 0.668±0.058 | 0.602±0.048 |
| LVPW; s (mm) | 1.217±0.061 | 1.023±0.061 | 0.7594±0.066** | 0.6332±0.064 |
| LVAW; d (mm) | 0.831±0.029 | 0.678±0.062 | 0.887±0.074 | 0.629±0.097 |
| LVAW; s(mm) | 1.364±0.0446 | 1.178±0.126 | 1.126±0.122 | 0.7636±0.119 |
| LV Vol; d (μl) | 45.38±3.533 | 55.93±4.655 | 68.31±3.925** | 81.32±3.699^##^ |
| LV Vol; s (μl) | 13.92±1.519 | 21.79±2.565 | 49.62±4.086** | 68.05±3.194^##^ |

Values are mean ± SEM, n = 6 per group. HR, heart rate; EF, ejection fraction; FS, fraction shortening; LV Mass, left ventricular mass; LVID, left ventricular internal diameter; LVPW, left ventricular posterior wall; LVAW, left ventricular anterior wall; LV Vol, left ventricular volume. **P* < 0.05, ***P* < 0.01 versus WT+sham; ^#^*P* < 0.05, ^##^*P* < 0.01 versus WT+I/R.

**Table S4.** Echocardiographic measurement of wild type (WT) and Pmsb8-KO mice injected with rAAV9-siRNA-control (rAAV9-siCon) or rAAV9-siRNA-Drp1 (siDrp1) following 24 h of I/R injury.

| Parameter | WT+rAAV9-siCon  I/R | Pmsb8-KO+ rAAV9-siCon  I/R | WT+ siDrp1  I/R | Pmsb8-KO+ siDrp1  I/R |
| --- | --- | --- | --- | --- |
| n | 6 | 6 | 6 | 6 |
| HR (bpm) | 490.3±8.456 | 496.8±7.079 | 500.5±6412* | 497.5±9.563 |
| EF (%) | 29.78±2.006 | 18.73±1.542 | 35.44±1.268* | 29.36±1.609^###^ |
| FS (%) | 13.19±0.886 | 9.112±1.011 | 16.69±0.693* | 13.54±0.795^##^ |
| LV Mass (AW)(mg) | 124.5±11.81 | 89.74±12.27 | 117.3±7.586 | 91.29±6.835 |
| LVID; d (mm) | 3.968±0.060 | 4.202±0.076 | 3.902±0.133 | 3.958±0.061^#^ |
| LVID; s (mm) | 3.452±0.090 | 3.904±0.088 | 3.305±0.138 | 3.480±0.102^#^ |
| LVPW; d (mm) | 0.7283±0.094 | 0.7262±0.019 | 0.8368±0.029 | 0.704±0.077 |
| LVPW; s (mm) | 0.9163±0.091 | 0.678±0.074 | 01.144±0.086 | 0.939±0.081^#^ |
| LVAW; d (mm) | 0.8082±0.067 | 0.642±0.064 | 0.828±0.075 | 0.702±0.053 |
| LVAW; s (mm) | 1.006±0.045 | 0.679±0.074 | 1.142±0.064* | 0.989±0.06^#^ |
| LV Vol; d (μl) | 69.50±3.291 | 77.44±3.668 | 57.95±1.718** | 69.81±2.758^##^ |
| LV Vol; s (μl) | 53.46±3.435 | 62.48±1.534 | 43.26±3.559* | 52.81±1.938^##^ |

Values are mean ± SEM, n = 6 per group. HR, heart rate; EF, ejection fraction; FS, fraction shortening; LV Mass, left ventricular mass; LVID, left ventricular internal diameter; LVPW, left ventricular posterior wall; LVAW, left ventricular anterior wall; LV Vol, left ventricular volume. **P* < 0.05, ***P* < 0.01 versus WT+rAAV9-siCon+I/R; ^#^*P* < 0.05, ^##^*P* < 0.01 versus Pmsb8-KO+ rAAV9-siCon+I/R.

**Table S5.** The primer sequences for qPCR analysis.

| **Gene name** | **Forward** | **Reverse** |
| --- | --- | --- |
| β1/Psmb6 | TCACTGCCAATGTGTCCTCG | CGTGGCAATGGTGAACTTGG |
| β2/Psmb7 | CTCGGCCCGGAAACACTTT | CGACCGAGATGCGTTCCTTA |
| β5/Psmb5 | CCTGGCCTTCAAGTTTCTCCA | CGACCGAGATGCGTTCCTTA |
| β1i/Psmb9 | AAGTCCACACCGGGACAAC | TCCCAGGATGACTCGATGGT |
| β2i/Psmb10 | TGGGACTAGCAGCAAAGACG | TGGCCTTGGTACCGGAAAAG |
| β5i/Pmsb8 | GCCAAGGAGTGCAGGTTGTAT | CCAAGGTCGTAGGCCTCTTC |
| Bax | TGGAGCTGCAGAGGATGATT | CTTGGATCCAGACAAGCAGC |
| Bcl-2 | CAGCCTGAGACAACCCAAT | TATAGTTCCACAAAGGCATCCCAG |
| Drp-1 | CAGCTGCACTGGCTTCATGACTC | GTCAACTTGCCATAAACCAGAG |
| Mfn-1 | ATGGCAGAAACGGTATCTCCA | GCCCTCAGTAACAAACTCCAGT |
| Mfn-2 | AGAACTGGACCCGGTTACCA | CACTTCGCTGATACCCCTGA |
| GADPH | GAAGGTCGGTGTGAACGGAT | ACTGTGCCGTTGAATTTGCC |
| ND-1 | \| CCCAGCCTACCAGATTTC \| \| --- \| | \| GGGTTGTATTGATGAGATTAGT \| \| --- \| |
| β-globin | \| GCTTCTGACACAACTGTGTTCACTAGC \| \| --- \| | \| CACCAACTTCATCCACGTTCACC \| \| --- \| |

**Note:** If necessary, we will provide the supplementary table about MS proteins analysis. The raw data were collected according to the LC-MS methods.

**Supplementary figure and figure legends**


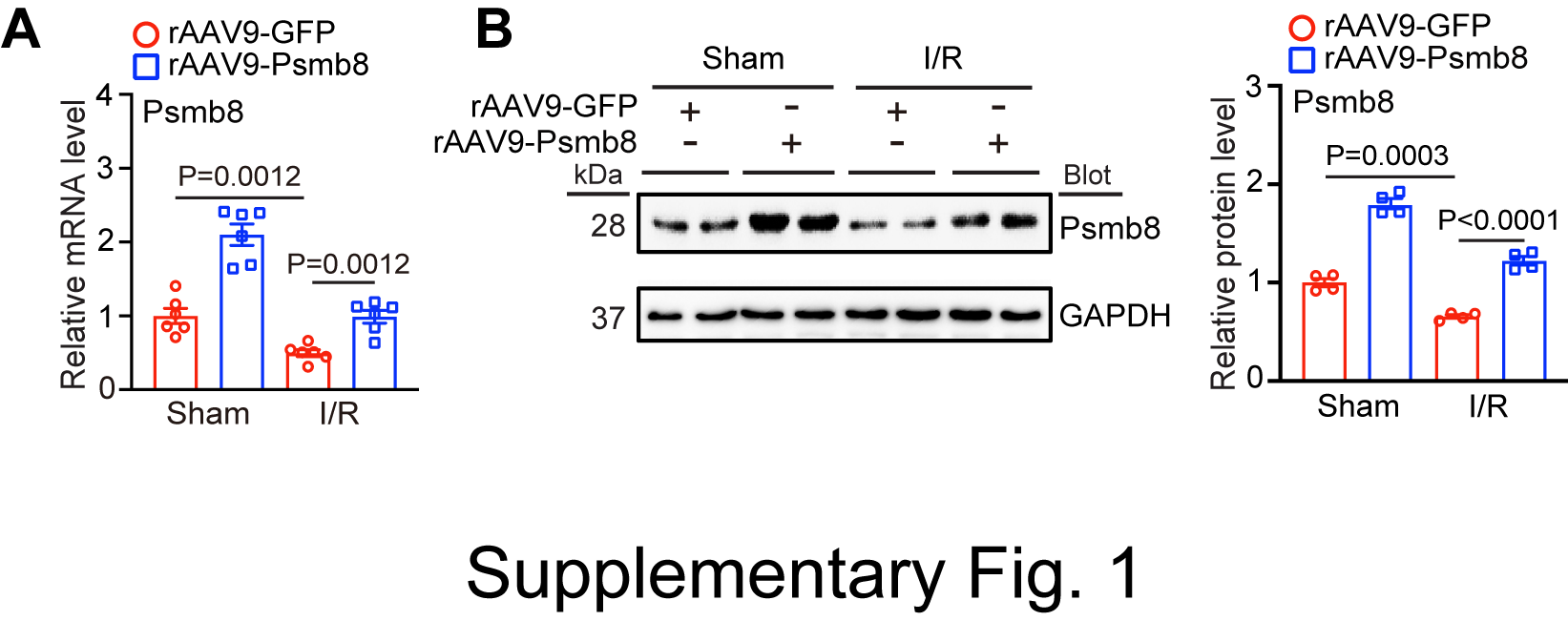


**Fig. S1. Analysis of the Pmsb8 mRNA and protein levels in the hearts of wild-type (WT) injected with rAAV9-Pmsb8 or rAAV9-GFP following 24 h of sham or I/R surgery.** **A** qPCR analysis of the Pmsb8 mRNA expression in the heart tissues of each group (n = 6). **B** Immunoblotting analysis of the Pmsb8 protein level in the heart tissues of each group, and quantification of the relative protein level (n = 4). Data are expressed as mean ± SEM, and n represents the number of samples per group.


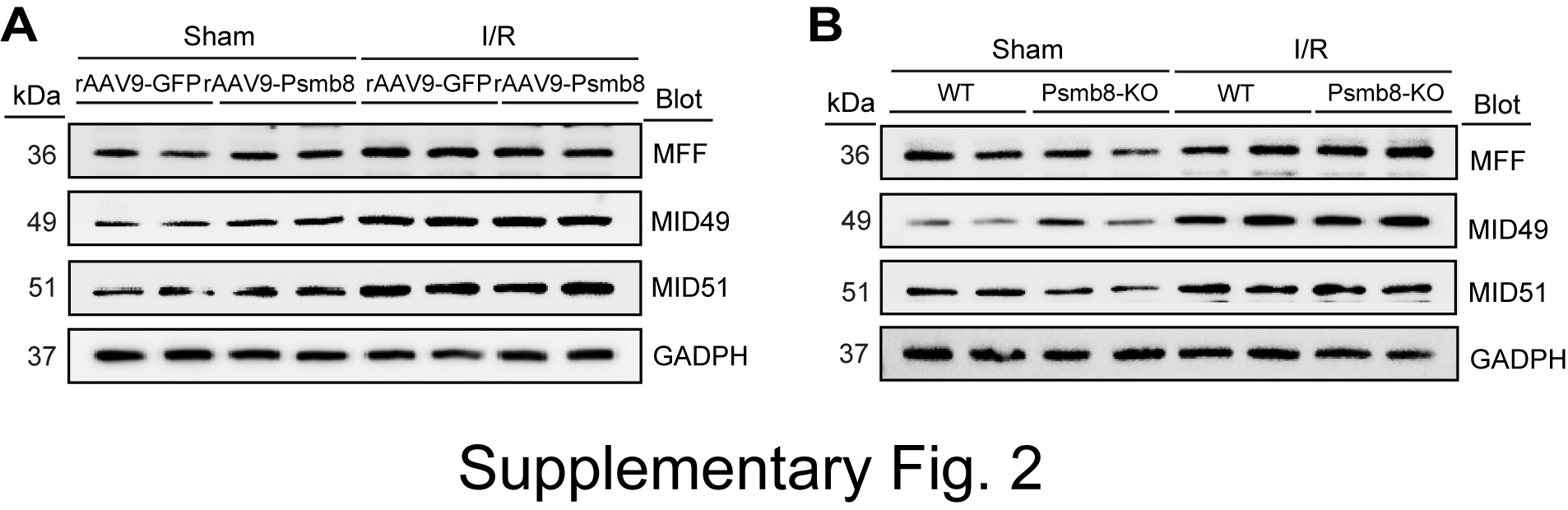


**Fig. S2. Analysis of Drp1 receptor protein levels in the hearts of Pmsb8 overexpressing and KO mice following 24 h of sham or I/R injury.** Immunoblotting analysis of MFF, MID49 and MID51 protein levels in the hearts of rAAV9-GFP- and rAAV9-Pmsb8-infected mice (**A**) or WT and Pmsb8-KO mice (**B**) following 24 h of sham or I/R injury.


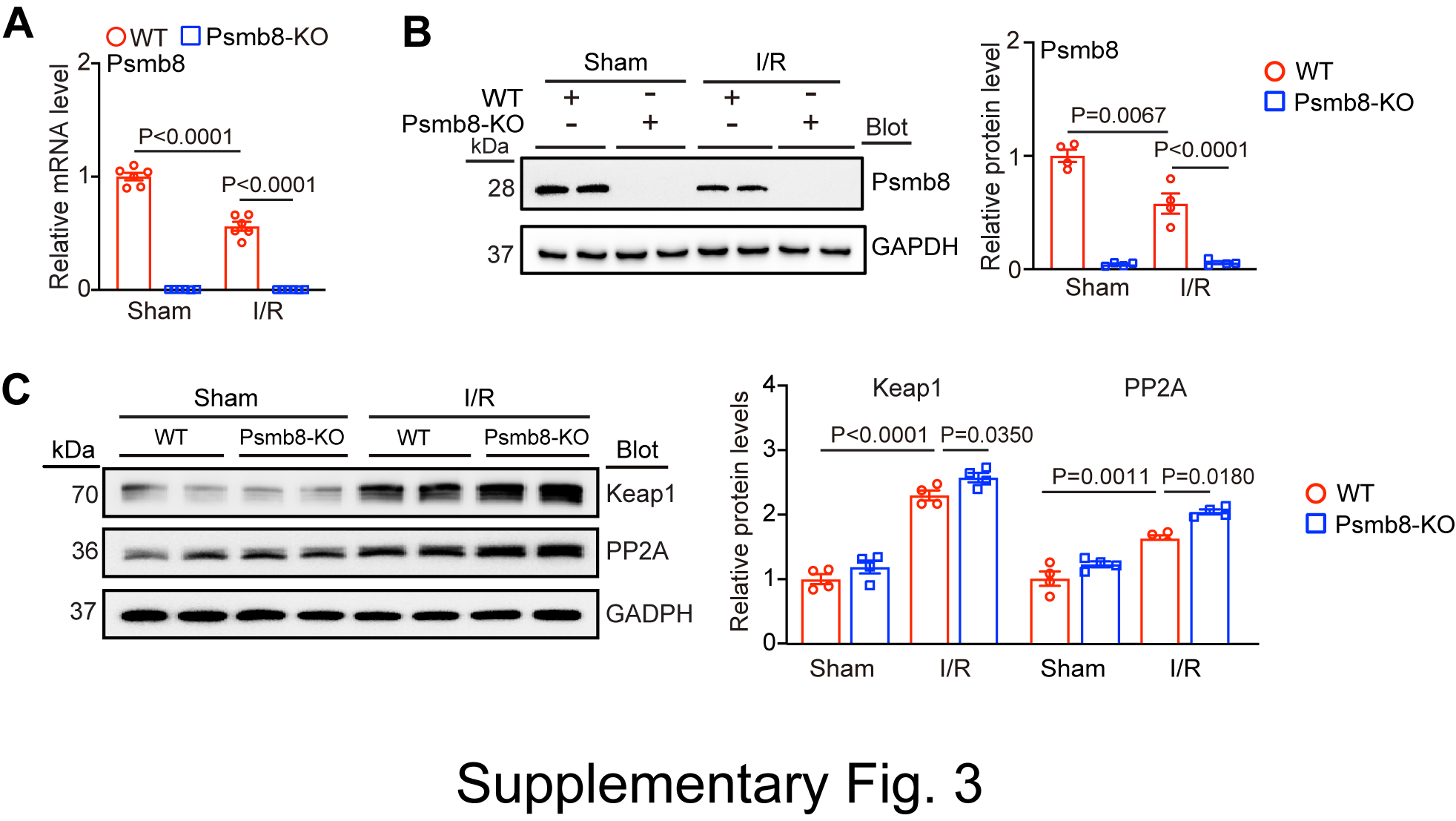


**Fig. S3. Analysis of the Pmsb8,Keap1 and PP2A levels in the hearts of wild-type (WT) and Pmsb8 knockout (KO) mice following 24 h of sham or I/R injury. A** qPCR analysis of the Pmsb8 mRNA expression in the heart tissues of each group (n = 6). **B** Immunoblotting analysis of the Pmsb8 protein level in the heart tissues and quantification of each group (n = 4). **C** Immunoblotting analysis of Keap1 and PP2A protein levels in the heart tissues of WT or Pmsb8-KO mice, and quantification of the relative protein level (n = 4). Data are expressed as mean ± SEM, and n represents the number of samples per group.


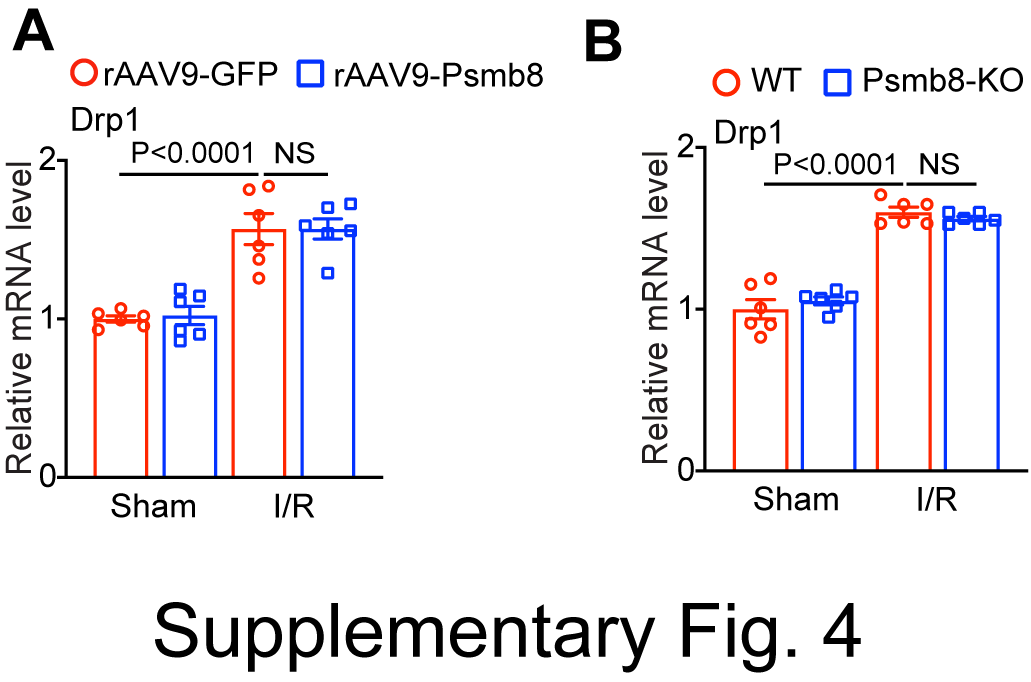


**Fig. S4. Analysis of the mRNA levels of Drp1 in the hearts of wild-type (WT), Pmsb8-KO, rAAV9-Pmsb8 or rAAV9-GFP-injected mice following sham or I/R injury.** **A** Male rAAV9-Pmsb8 or rAAV9-GFP-injected mice were subjected to sham or I/R surgery for 24 h. qPCR analysis of the mRNA levels of Drp1, Mfn1, and Mfn2 in the heart tissues (n = 6). **B** Male wild-type (WT) and Pmsb8-KO mice were subjected to sham or I/R surgery for 24 h. qPCR analysis of the mRNA levels of Drp1 in the heart tissues of each group (n = 6). Data are expressed as mean ± SEM, and n represents the number of samples per group.
