# Supplementary material for: Psmb8 inhibits mitochondrial fission and alleviates myocardial ischaemia/reperfusion injury by targeting Drp1 degradation
Source: Cell Death Dis. 2024 Nov 8;15(11):803. doi: 10.1038/s41419-024-07189-1 (PMC11549449; doi:10.1038/s41419-024-07189-1)

Figure 1B

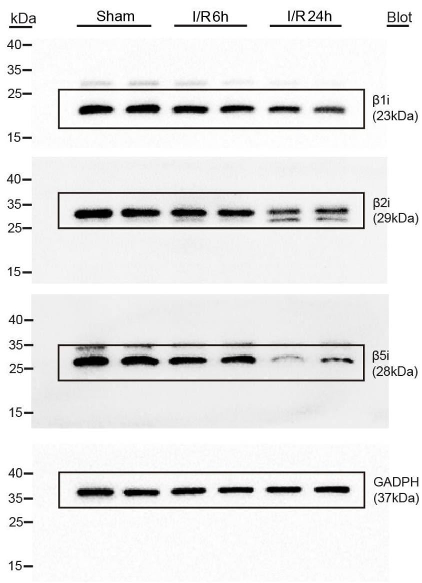

Figure 1C

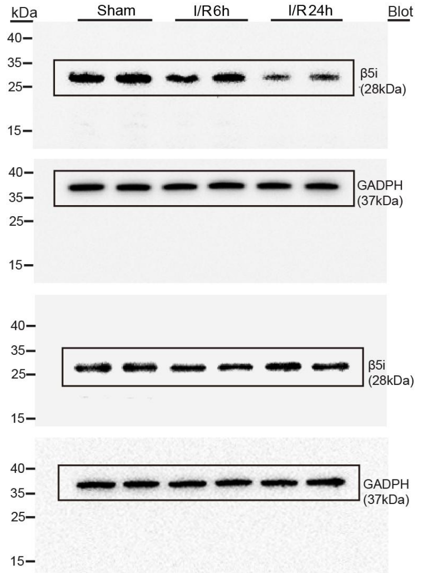

Figure 2D

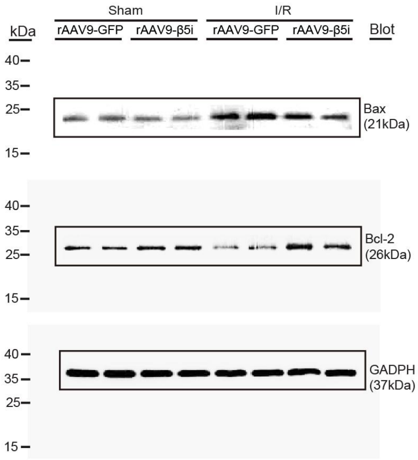

Figure 3G

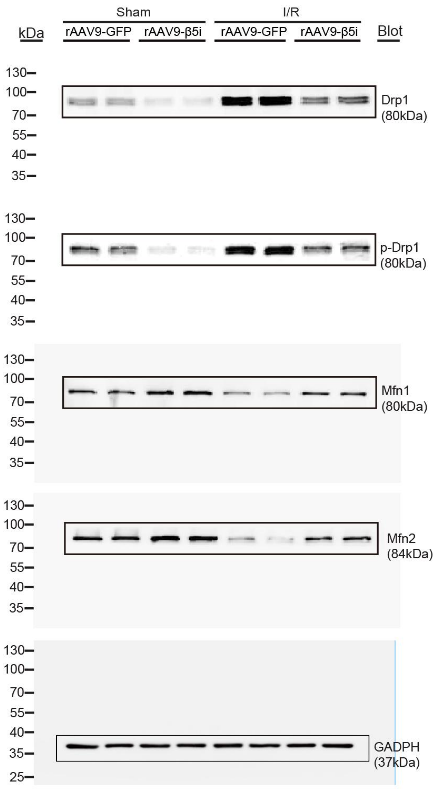

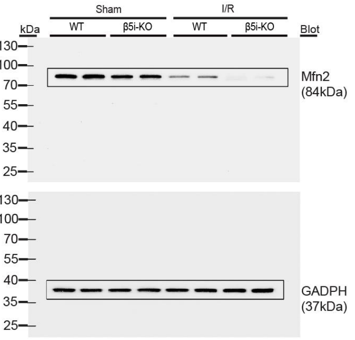

Figure 4D

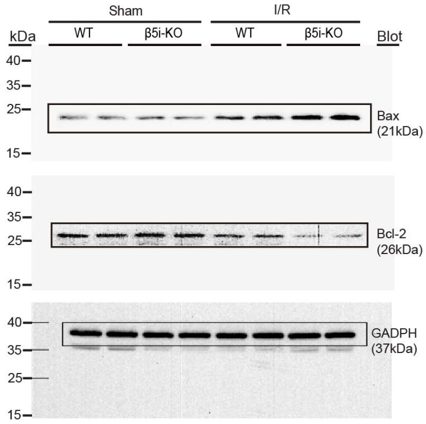

Figure 4H

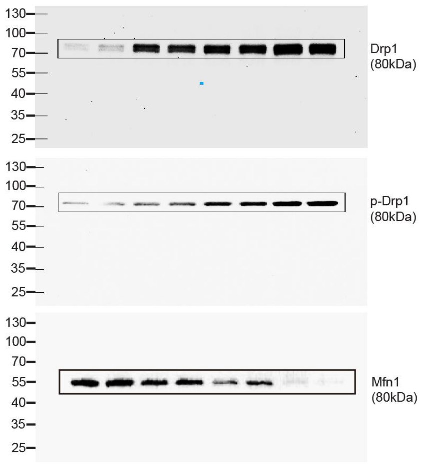

Figure 5E

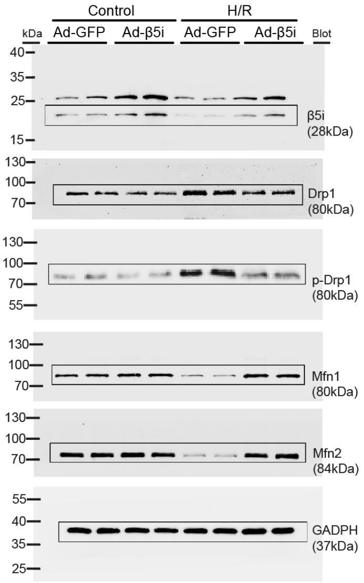

Figure 5F

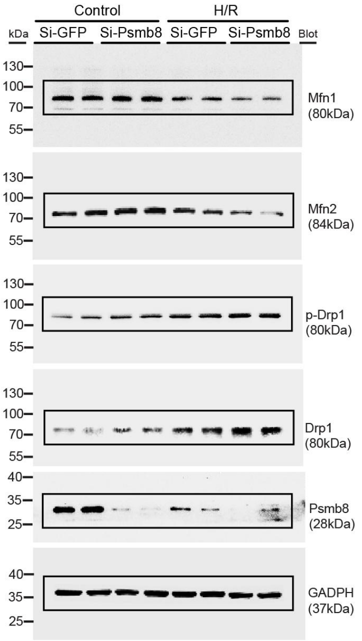

Figure 5F

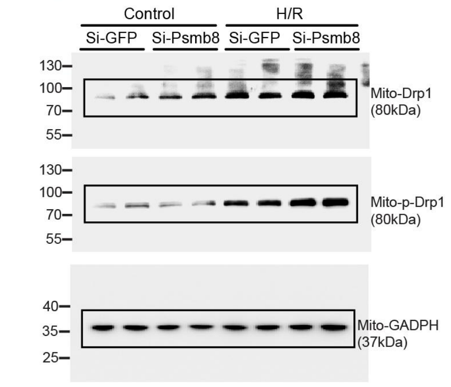

Figure 5G

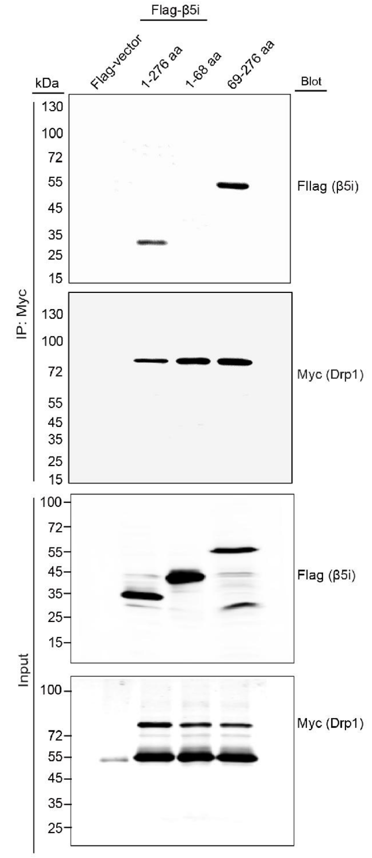

Figure 6E

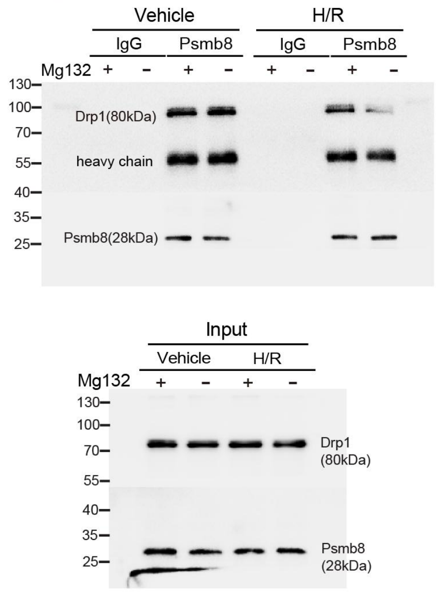

Figure 6H

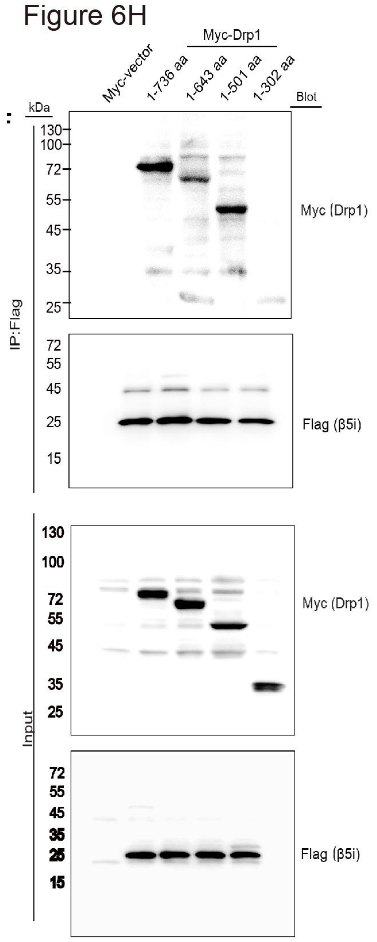

Figure 6D

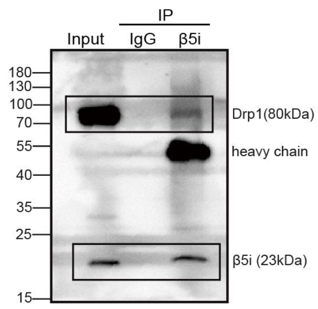

Figure 7A

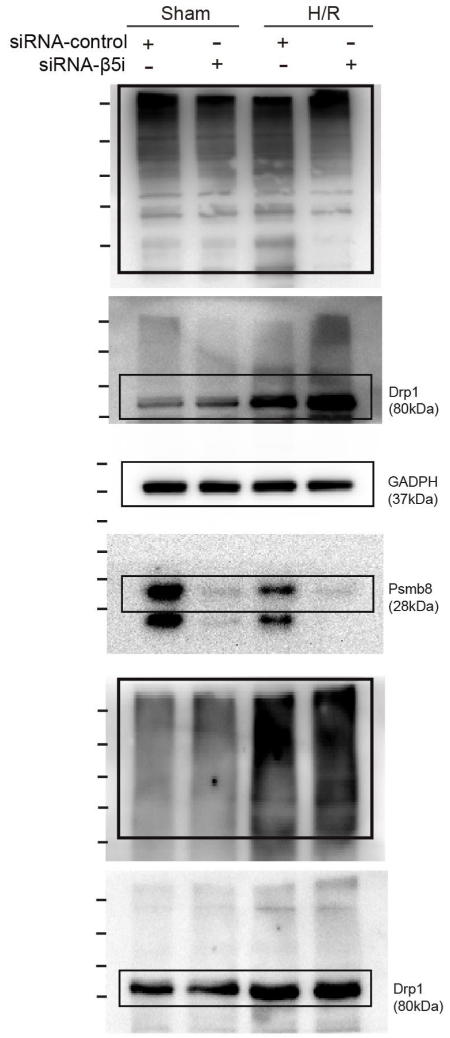

Figure 7B

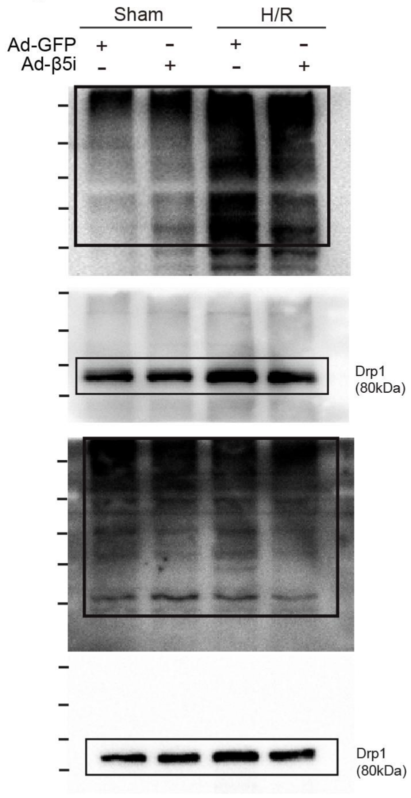

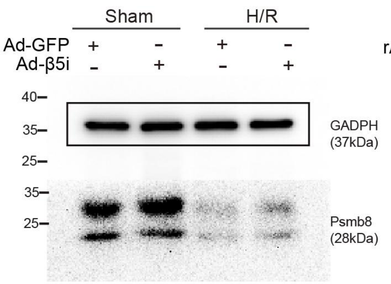

Figure 7C

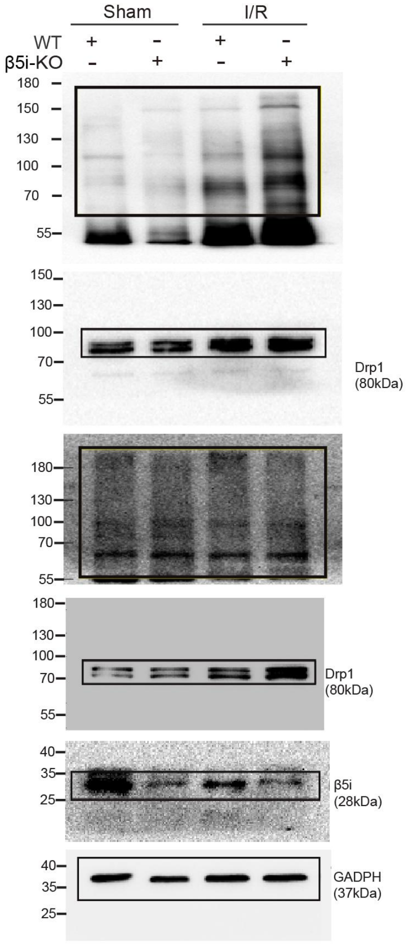

Figure 7D

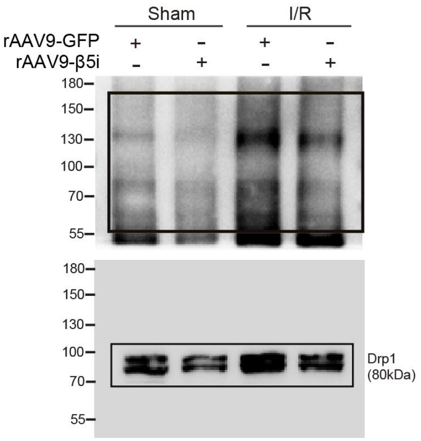

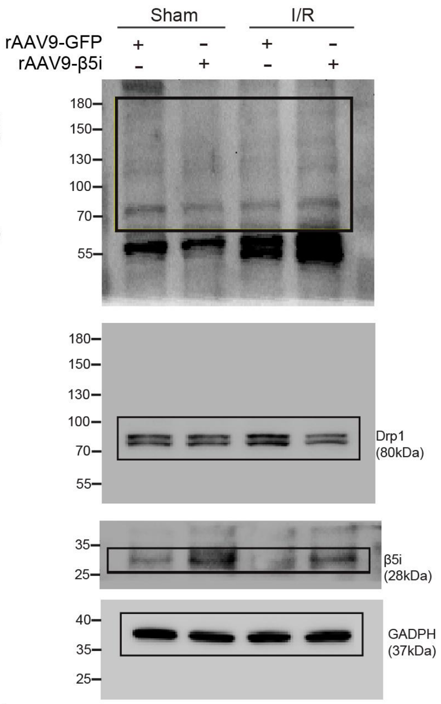

Figure 7E

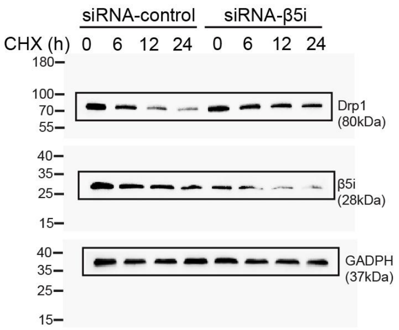

Figure 7F

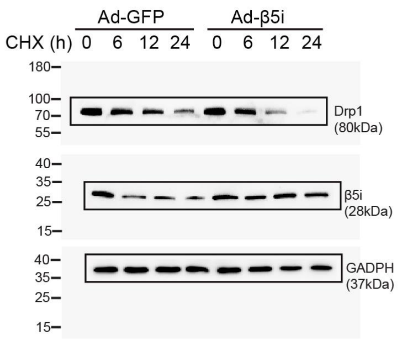

Figure 8A

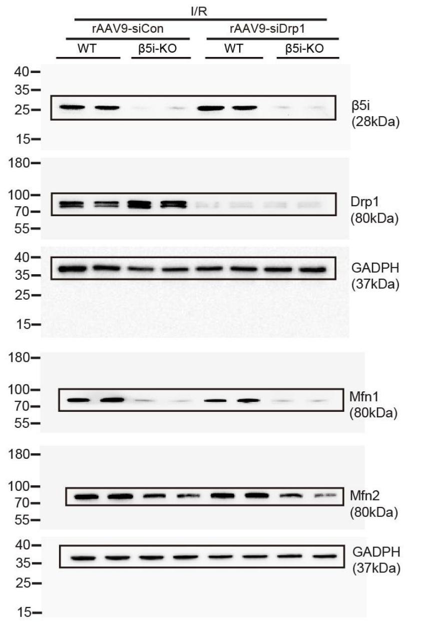

Figure S1B

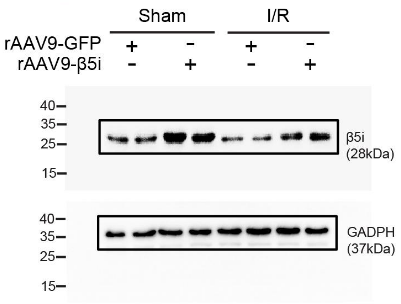

Figure S2A

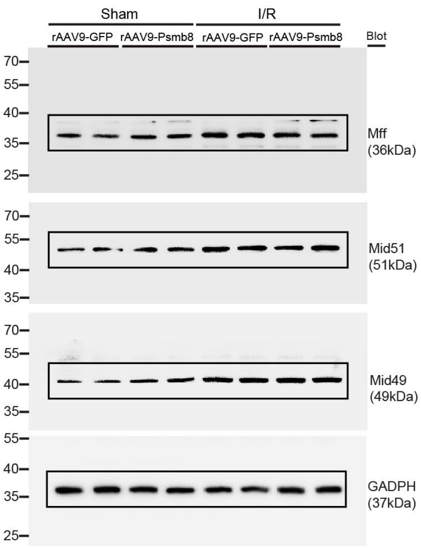

Figure S2B

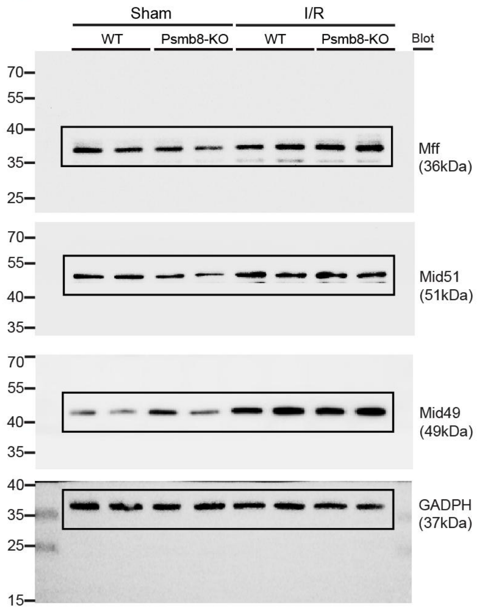

Figure S3B

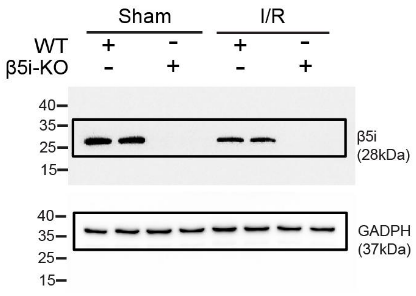

Figure S3C

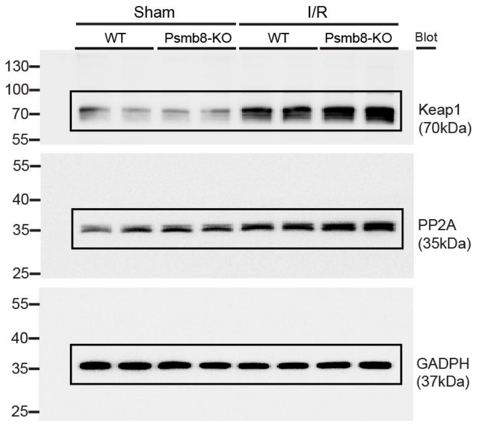

Supplement: Supplementary file 4 — Supplementary Data-WB original figures [file 41419_2024_7189_MOESM4_ESM.pdf]
